# Childhood neurodevelopment after prescription of maintenance methadone for opioid dependency in pregnancy: a systematic review and meta‐analysis

**DOI:** 10.1111/dmcn.14117

**Published:** 2018-12-03

**Authors:** Victoria J Monnelly, Ruth Hamilton, Francesca M Chappell, Helen Mactier, James P Boardman

**Affiliations:** ^1^ MRC Centre for Reproductive Health University of Edinburgh Edinburgh UK; ^2^ Department of Clinical Physics and Bioengineering Royal Hospital for Children Glasgow UK; ^3^ Centre for Clinical Brain Sciences University of Edinburgh Edinburgh UK; ^4^ Princess Royal Maternity Glasgow UK

## Abstract

**Aim:**

To systematically review and meta‐analyse studies of neurodevelopmental outcome of children born to mothers prescribed methadone in pregnancy.

**Method:**

MEDLINE, Embase, and PsycINFO were searched for studies published from 1975 to 2017 reporting neurodevelopmental outcomes in children with prenatal methadone exposure.

**Results:**

Forty‐one studies were identified (2283 participants). Eight studies were amenable to meta‐analysis: at 2 years the Mental Development Index weighted mean difference of children with prenatal methadone exposure compared with unexposed infants was −4.3 (95% confidence interval [CI] −7.24 to −1.63), and the Psychomotor Development Index weighted mean difference was −5.42 (95% CI −10.55 to −0.28). Seven studies reported behavioural scores and six found scores to be lower among methadone‐exposed children. Twelve studies reported visual outcomes: nystagmus and strabismus were common; five studies reported visual evoked potentials of which four described abnormalities. Factors that limited the quality of some studies, and introduced risk of bias, included absence of blinding, small sample size, high attrition, uncertainty about polydrug exposure, and lack of comparison group validity.

**Interpretation:**

Children born to mothers prescribed methadone in pregnancy are at risk of neurodevelopmental problems but risk of bias limits inference about harm. Research into management of opioid use disorder in pregnancy should include evaluation of childhood neurodevelopmental outcome.

**What this paper adds:**

- Children born to opioid‐dependent mothers prescribed methadone are at risk of neurodevelopmental impairment.
- Exposed infants have lower Mental Development Index and Psychomotor Development Index scores than unexposed children.
- Atypical visual evoked potentials, strabismus, and nystagmus have increased prevalence.
- Estimates of impairment may be biased by intermediate to poor quality evidence.

## What this paper adds

- Children born to opioid‐dependent mothers prescribed methadone are at risk of neurodevelopmental impairment.
- Exposed infants have lower Mental Development Index and Psychomotor Development Index scores than unexposed children.
- Atypical visual evoked potentials, strabismus, and nystagmus have increased prevalence.
- Estimates of impairment may be biased by intermediate to poor quality evidence.

**Editor's Choice**

My Editor's Choice for the July 2019 issue is this systematic review. It highlights neurodevelopmental outcomes of children who had antenatal exposure to methadone given to their mother as maintenance treatment (as well as other, less documented risk factors). The surge in opiod dependence in the USA and other countries, particularly in young adults, raises specific concerns for the children's development.

This article is commented on by Skranes on page 738 of this issue.

This article's abstract has been translated into Spanish and Portuguese.

Follow the links from the abstract to view the translations.

Video Podcast: https://www.youtube.com/watch?v=Ixo12Fg0FJs

## Resumen

**Objetivo:**

Revisar sistemáticamente y realizar un meta‐análisis de estudios sobre el resultado del desarrollo neurológico de los niños nacidos de madres a quienes se les recetó metadona durante el embarazo.

**Metodología:**

Se realizó una búsqueda en MEDLINE, Embase, y PsycINFO de estudios publicados desde el año 1975 al 2017 que informaran sobre el resultado del desarrollo neurológico de niños que hubieran tenido exposición prenatal a la metadona.

**Resultados:**

Se identificaron 41 estudios (2283 participantes). Ocho estudios se pudieron someter al meta‐análisis: a los dos años de edad los niños con exposición prenatal a la metadona mostraron una diferencia de medias ponderada de ‐4,3 (95% intervalo de confianza [IC] −7,24 to −1,63) en el Índice de Desarrollo Mental (Mental Development Index) en comparación con los niños no expuestos. En el Índice de Desarrollo Psicomotor (Psychomotor Development Index) la diferencia de medias ponderada fue −5,42 (95% CI −10,55 to −0,28). 7 estudios mostraron las puntuaciones comportamentales y 6 de ellos encontraron puntuaciones más bajas entre los niños expuestos a la metadona. Doce estudios informaron sobre los resultados a nivel visual: el nistagmo y el estrabismo fueron comunes; 5 estudios informaron sobre los potenciales evocados visuales, de los cuáles cuatro describieron anormalidades. Los factores que limitaron la calidad de algunos estudios e introdujeron el riesgo de sesgo, incluyeron la ausencia de cegamiento, el pequeño tamaño de la muestra, el alto desgaste, la incertidumbre acerca de la exposición a varias drogas y la falta de validez del grupo de comparación.

**Interpretación:**

Los niños nacidos de madres a quienes se les recetó metadona durante el embarazo se encuentran en riesgo de sufrir problemas de desarrollo neurológico, pero el riesgo de sesgo limita la inferencia sobre el daño. La investigación sobre el manejo del trastorno por uso de opioides en el embarazo debe incluir la evaluación del resultado del desarrollo neurológico infantil.

## Resumo

**Objetivo:**

Revisar sistematicamente e metanalisar os estudos com resultados do neurodesenvolvimento de crianças nascidas de mães que tiveram prescrição de metadona na gestação.

**Método:**

MEDLINE, Embase, e PsycINFO foram pesquisadas por estudos publicados de 1974 a 2017 relatando resultados do neurodesenvolvimento em crianças expostas a metadona no período pré‐natal.

**Resultados:**

Quarenta e um estudos foram identificados (2.283 participantes). Oito estudos foram possíveis de incluir na metanálise: aos 2 anos a diferença na média ponderada do Índice de Desenvolvimento Mental de crianças expostas a metadona pré‐natal comparadas com as não expostas foi −4,3 (intervalo de confiança [IC a 95%] −7,24 a −1,63), e a diferença na média ponderada do Índice de Desenvolvimento Psicomotor foi −5,42 (IC 95% −10,55 a −0,28). Sete estudos relataram escores comportamentais e seis encontraram escores menores entre crianças expostas a metadona. Doze estudos relataram resultados visuais: nistagmo e estrabismo foram comuns; cinco estudos reportaram potenciais evocados visuais, dos quais quatro descreveram anormalidades. Fatores que limitaram a qualidade de alguns estudos e introduziram risco de viéis incluíram falta de cegamento, reduzido tamanho amostral, desgaste alto, incerteza sobre exposição a outras drogas, e falta de validade por grupo de comparação.

**Interpretação:**

Crianças nascidas de mães que receberam prescrição de metadona na gestação apresentam risco para problemas neurodesenvolvimentais, mas o risco de viés limita as inferências sobre o dano. Pesquisas sobre o manejo do uso de opióides na gestação devem incluir a avaliação do resultado do neurodesenvolvimento na infância.

**Abbreviations**
MAT: Medically assisted treatment
MDI: Mental Development Index
NAS: Neonatal abstinence syndrome
PDI: Psychomotor Development Index
VEP: Visual evoked potential
WMD: Weighted mean difference

Opioid use, both prescribed and illicit, has been increasing globally since 2007. Past‐year prevalence of heroin use has almost doubled since 2007, and the rate of increase is higher among women compared with men. In the USA, the average rate of past‐year heroin use between 2013 and 2015 was 2.0 per 1000 women,^1^ and since 2000 there has been an almost fivefold increase in the prevalence of neonatal abstinence syndrome (NAS), a drug withdrawal syndrome commonly used as a proxy for opioid exposure during pregnancy.^2^ It is estimated that in the current opioid crisis up to 14.4% of pregnant women have opioid prescriptions dispensed during pregnancy.^3^

Pregnant women who use heroin are recommended medically assisted treatment (MAT) with an opioid substitute such as methadone as part of a comprehensive antenatal care plan because it is associated with improved use of antenatal services, reduced use of heroin during pregnancy, and reduced risk of preterm delivery, when compared with no treatment.^4^, ^5^, ^6^, ^7^, ^8^, ^9^ Fetal benefits of MAT include improved growth^7^, ^10^ and less risk of intrauterine death.^8^

Methadone is a synthetic long acting μ‐opioid agonist which freely crosses the placenta; despite the potential for methadone to affect the developing fetal brain, this treatment was introduced into practice without a randomized controlled study of childhood neurodevelopmental outcome. Preclinical studies suggest that exogenous opioids may exert pleiotropic harmful effects on the central nervous system,^11^, ^12^, ^13^ and diffusion magnetic resonance imaging studies show that the tract tissue microstructure of white matter (fractional anisotropy) is altered in neonates exposed prenatally to methadone.^14^, ^15^ Improved understanding of the neurodevelopmental outcome of children born to opioid‐dependent mothers and exposed prenatally to methadone is essential to inform management of their mothers during pregnancy.^16^, ^17^, ^18^ The issue is prescient because the optimal methadone dose regimen is uncertain,^19^ and alternative opioids such as buprenorphine may have a different risk profile for neonatal outcome,^16^, ^17^, ^20^ leading to equipoise about the optimal MAT strategy.

The aims of this study were to perform a systematic review of published literature on childhood neurodevelopmental outcomes after prescription of maintenance methadone in pregnancy, and to undertake a meta‐analysis of studies that used a common assessment tool.

## Method

The study protocol was registered with the international prospective register of systematic reviews (PROSPERO), registration number CRD42017063987 (https://www.crd.york.ac.uk/prospero/). Methodology is reported according to the Preferred Reporting Items for Systematic Reviews and Meta‐Analyses (PRISMA) statement.^21^

We included all studies that reported neurodevelopmental outcome, including visual development, of children whose opioid‐dependent mothers were prescribed methadone during pregnancy. There was no language restriction. Exclusion criteria were prescription of alternative opioid substitutes during pregnancy and studies reporting only neonatal neurodevelopment.

Two reviewers (VJM, RH) independently searched MEDLINE, Embase, and PsycINFO for studies published between 1975 and 2017. Medical Subject Headings terms used were ‘methadone’ and ‘prenatal’ or ‘prenatal exposure’ or ‘prenatal drug exposure’ or ‘prenatal exposure delayed effects’ or ‘in utero’. Bibliographies of primary studies and review articles meeting the inclusion criteria were searched manually to identify further eligible studies.

Three reviewers (VJM, RH, HM) independently screened titles and abstracts to identify potentially eligible studies. Where necessary to determine eligibility, full text was retrieved and reviewed. Duplication was avoided if it was clear that the same cohort was reported in two publications; where more than one publication for a study was retrieved, only the report that contained the maximum data points was included.

Four reviewers (VJM, RH, HM, JPB) independently extracted data from included studies using a standardized template. Extracted information included study setting, design, population and participant demographics, details of methadone exposure if available, control conditions, recruitment and completion rates, age at outcome measurement, assessment tool, and outcome of assessment. Data were extracted from each study by two reviewers independently, and templates were combined to ensure complete data collection. Disagreements were resolved through discussion.

A quality assessment instrument was developed using the Grading of Recommendations Assessment Development and Evaluation Guidelines^22^, ^23^, ^24^ to provide a structured scoring system that aimed to describe quality and sources of bias in studies of neurodevelopment after prenatal drug exposure. It incorporated objective criteria about study design, sample size and characteristics, use of validated outcome measures, risk of bias (blinding, confounding, attrition), and data analysis. Each study was assessed independently by two reviewers (VJM, JPB) and scored as good (A, 6.5–8), intermediate (B, 3.5–6), or poor (C, 1–3) quality (Table SI, online supporting information).

### Statistical analysis

Where studies used the same assessment tool for any outcome domain, quantitative data were pooled in random effects meta‐analysis using R software, version 3.2.2 (K Hornik; R Foundation for Statistical Computing, Vienna, Austria; https://cran.r-project.org/) with mean difference weighted by the inverse of the variance.^25^ Effect sizes were expressed as weighted mean differences (WMD) and their 95% confidence intervals (CI). For longitudinal studies, data for assessments at 6 months and at 2 years were analysed. Heterogeneity was assessed using the standard *I* ^2^ and *τ* statistics and graphically using forest plots. Where statistical pooling was not possible, data were collated in tables for outcomes across two domains (neurodevelopmental and visual development), and statements generated to represent the body of literature reviewed.

## Results

### Characteristics of included studies

Forty‐one eligible studies were identified, including a total of 1441 methadone‐exposed children and 842 unexposed children (Fig. S1, online supporting information). Twenty‐nine studies reported neurodevelopmental outcome (1247 methadone‐exposed vs 740 unexposed children), eight of which were amenable to meta‐analysis; 12 reported visual outcome (275 methadone‐exposed vs 128 unexposed). There were no randomized trials.

Only one study fulfilled criteria for good quality;^26^ 26 (63%) studies were intermediate and 14 (34%) were poor quality (Table SI). Study deficiencies included lack of blinding, small sample size, high attrition rates, and lack of comparison group validity. Twenty‐four of 41 studies reported information about polydrug use during pregnancy, and 20 studies provided information about methadone dose exposure.

Thirty‐three of 41 studies reported infants receiving pharmacological treatment of NAS; in 19 of these the treatment regimen was described, with the most frequently used drugs being morphine, phenobarbital, benzodiazepines, or a combination. Sixteen studies stated that infants born preterm were included, while only seven explicitly excluded infants born before 36 weeks’ gestation. In 18 studies, it could not be determined whether infants born preterm were included.

### Neurodevelopmental outcome

Of 29 studies reporting neurodevelopmental outcome,15 used the original Bayley Scales of Infant Development.^27^ Five of these 15 studies had no comparison individuals,^28^, ^29^, ^30^, ^31^, ^32^ one did not report a measure of variance,^33^ and one assessed children at 9 months only;^34^ leaving eight studies that were eligible for meta‐analysis of neurodevelopmental outcome based on the Bayley Scales of Infant Development (Table 1).

**Table 1:** Summary of the eight studies included in the meta‐analysis

Study	Quality rating^a^	Methadone‐exposed	Unexposed	Age^b^	Drug information^c^	Assessment tool	Main findings^d^ (results appear as methadone vs unexposed)	Comments^e^
Strauss et al.^35^,^f^	B	25	26	3mo	No dosing information No drug screening or polydrug information	BSID (MDI, PDI) at all ages	6mo^f^: MDI 115.7 (16.8) vs 114.3 (20.9), PDI 109.4 (12.2) vs 111.7 (14.5)	Original cohort 60 methadone‐exposed infants vs 53 unexposed infants (no information on matching); data reported only for infants who underwent BSID at all three time points. Attrition rate at 6mo was 58.4% vs 51%. One case of SIDS in the methadone‐exposed group. Gestational age at birth not stated. Assessor blinding not stated. No information about NAS or treatment.
		25	26	6mo^f^				
		25	26	1y				
Kaltenbach et al.^40^,^g^	B	26	27	1y	Mean dose for 1y cohort: 30; mean dose for 2y cohort: 18 No drug screening or polydrug information	BSID (MDI) at all ages	2y^g^: MDI 90.88 (8.26) vs 94.62 (11.93) ns	Original cohort 43 methadone‐exposed infants vs 51 unexposed (matched for maternal SES, ethnicity and medical conditions). Attrition rate at 2y 60.5% vs 53%. Assessors blinded to group. 62% 1y‐olds and 67% 2y‐olds had been treated for NAS in the neonatal period. No pharmacological agent stated
		17	24	2y^g^				
Chasnoff et al.^36^,^f^,^g^	B	31	34	3mo	Mean dose 14.6±10.2 (5–40) Maternal interview and urine screening; 4 out of 31 used drugs in addition to heroin during pregnancy	BSID (MDI, PDI) at all ages	6mo^f^: MDI 105.9 (12.4) vs 111.0 (12.3); PDI 103.9 (9.0) vs 107.6 (15.1) 2y^g^: MDI 97.5 (16.1) vs 96.2 (15.9); PDI 99.3 (16.9) vs 98.2 (8.9) No *p*‐values stated	Original cohort 39 methadone exposed vs 34 unexposed (matched for maternal age, education, gravidity, and smoking). Attrition rate at 6mo was 66.7% vs 14.7%; at 2y attrition rate was 84.6% vs 58.8%. All infants were born at term. Assessor blinding not stated. No information about NAS or treatment.
		13	29	6mo^f^				
		11	27	1y				
		6	14	2y^g^				
Rosen and Johnson^37^,^f^,^g^	B	41	23	6mo^a^,^f^	42 (mean of original cohort) Maternal urine screening; 56% of original cohort polydrug use (BDZ, opiates, cocaine, barbiturates, TCA) 15% reported ‘mod‐severe’ alcohol intake	BSID (MDI, PDI) at 6mo, 12mo, 18mo, and 2y; M‐P at 3y	6mo^f^: MDI 95 (2.5) vs 100.7 (4.2), ns, PDI 101 (2.8) vs 105.1 (2.9), ns 2y^g^: MDI 90.4 (2.6) vs 96.9 (3.1) ns PDI 99.1 (2.7) vs 108 (2.7), *p*=0.05 All scores are mean (standard error)^h^	Original cohort 61 methadone‐exposed infants vs 32 unexposed infants (matched for maternal ethnicity, SES, infant sex, birthweight, and gestational age). Attrition rate at 6mo was 32.8% vs 28.1%; at 2y was 44.3% vs 31.3%. Both groups included infants born preterm. Assessor blinding not stated. 75% methadone‐exposed had NAS; number treated pharmacologically not stated
		41	22	1y				
		38	23	18mo				
		34	22	2y^a^,^g^				
		39	21	3y				
Kaltenbach and Finnegan^38^,^f^,^g^	B	27	17	6mo^f^	Mean dose 38.42 No drug screening or polydrug information	BSID (MDI) at 6mo, 1y, and 2y; MSCA (GCI) at 3y 6mo–4y 6mo	6mo^f^: MDI 107.9 (12.23) vs 105.6 (7.31), no *p‐*value 2y^g^: MDI 100.9 (18.04) vs 103.9 (11.49) no *p*‐value	No information about original cohort, therefore attrition rates unknown. Unexposed group were matched for maternal ethnicity and SES. Mean gestational age infants not stated. Blinding of assessors not stated. 92% were treated for NAS; pharmacological agent not stated.
		27	17	1y				
		27	17	2y^g^				
		27	17	3y 6mo–				
		27	17	4y 6mo				
Wilson^41^,^g^	B	33	54	9mo	No dosing information Maternal urine screening; 93% used psychoactive drugs	BSID (MDI) at 9mo, 18mo, and 2y^c^	2y^g^: MDI 88.8 (15.5) vs 90.2 (14.6) ns	Original cohort 39 methadone‐exposed vs 57 unexposed infants (matched for maternal age, ethnicity, SES, and marital status). Attrition rate at 2y was 18% vs 16%. Mean gestational age not stated for either group. Assessor blinding not stated. 87% original cohort treated for NAS, pharmacological agent not stated.
		29	42	18mo				
		32	48	2y^g^				
		26	41	3–5y				
		12	12	6–11y				
van Baar^39^, ^f^,^g^	B	21	37	6mo^f^	No dosing or screening information; Original cohort; six IV drug users; 94% used multiple drugs; 60% use cocaine	BSID (MDI, PDI, NDI) at all ages WWPA^f^ at 18mo (*n*=14), 2y (*n*=16), and 2y 6mo (*n*=15)	6mo^f^; MDI 103 (12) vs 107 (13), PDI 116 (18) vs 114 (21), NDI 105 (13) vs 109 (14) 2y^g^: MDI 86 (15) vs 98 (16) *p*<0.05 PDI 102 (16) vs 100 (18) ns, NDI 93 (16) vs 102 (22)	Original cohort 35 methadone‐exposed vs 37 unexposed infants (not matched). Attrition rate at 6mo was 19.2% vs 0% and at 2y was 19.3% vs 8.1%. Assessor blinding not stated. 28 out of 35 (80%) were treated for NAS, pharmacological agent not stated.
		21	34	1y				
		18	34	18mo				
		21	34	2y^g^				
		19	34	2y 6mo				
Hans and Jeremy^42^, ^g^	B	33	45	4mo	Mean <20 (range 3–40) Maternal interview and MUS; 13 out of 33 cocaine, 18 out of 33 cannabis, 11 out of 33 alcohol use	BSID (MDI, PDI) at all ages	2y^g^: MDI 92 (12.7) vs 96 (12.3); PDI 100 (14.2) vs 108 (14.9)	Original cohort 47 methadone‐exposed vs 45 unexposed infants (matched for maternal age, SES, and IQ). Attrition rate 29.8% vs 0%. Assessors blinded to group. No information about NAS or treatment.
		33	45	8mo				
		33	45	1y				
		33	45	18mo				
		33	45	2y^g^				

^a^Quality rating: A, good; B, intermediate; C, poor; based on modified Grading of Recommendations Assessment Development and Evaluation (GRADE) criteria (Table SI, online supporting information). ^b^Age expressed in months (mo) or years (y). ^c^Drug information includes mean daily methadone dose (in milligrams), maternal urine screening, and/or infant urine screening for drug exposure and information on maternal polydrug use (defined as methadone plus any other drug use during pregnancy, excluding tobacco), where these are reported. Unless otherwise stated, all information in this column refers to methadone‐exposed group only. ^d^Full details from these studies is available in Table SII (online supporting information). Scores are presented as mean values (standard deviation) unless otherwise stated. ^e^Comments include information on attrition, matching, gestation, blinding, proportion of infants treated for NAS, and pharmacological treatment for NAS, where provided in the original study. ^f^Studies included in meta‐analysis at 6mo. ^g^Studies included in meta‐analysis at 2y. ^h^Standard errors were converted to standard deviations for the meta‐analysis. BSID, Bayley Scales of Infant Development; MDI, Mental Developmental Index; PDI, Psychomotor developmental Index; SIDS, sudden infant death syndrome; NAS, neonatal abstinence syndrome; SES, socio‐economic status; BDZ, benzodiazepine; TCA, tricyclic antidepressant; M‐P, Merrill‐Palmer Scale; MSCA, McCarthy Scales of Childhood Abilities; GCI, General Cognitive Index; ns, not significant; IV, intravenous; NDI, non‐verbal developmental index; WWPA, Werry‐Weiss Peters Activity Scale; MUS, maternal urine screening.

**Figure 1:**
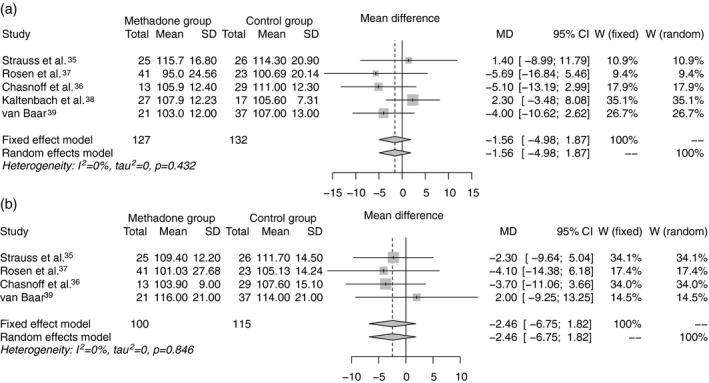
(a) Weighted mean difference in Mental Developmental Index and (b) Psychomotor Developmental Index of the Bayley Scales of Infant Development at age 6 months between methadone‐exposed and unexposed infants.

Five studies reported Mental Development Index (MDI) at 6 months of age,^35^, ^36^, ^37^, ^38^, ^39^ and four of these reported Psychomotor Development Index (PDI)^35^, ^36^, ^37^, ^39^ (Fig. 1). Studies were all of intermediate quality with attrition rates ranging from 31% to 70%; three studies described maternal methadone doses, and gestational age was variably reported. For both MDI and PDI at 6 months, the difference in exposed versus non‐exposed infants was marginal and 95% CIs included the possibility of no difference: MDI, WMD of −1.56 (95% CI −4.98 to 1.87; Fig. 1a); PDI, WMD of −2.46 (95% CI −6.75 to 1.82; Fig. 1b).

**Figure 2:**
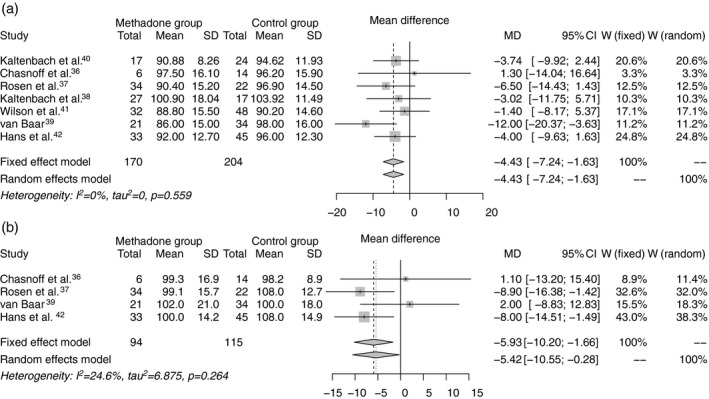
(a) Weighted mean difference in Mental Developmental Index and (b) Psychomotor Developmental Index of the Bayley Scales of Infant Development at age 18 to 24 months between methadone‐exposed and unexposed infants.

Seven studies reported MDI at 2 years of age,^36^, ^37^, ^38^, ^39^, ^40^, ^41^, ^42^ four of which reported PDI^36^, ^37^, ^39^, ^42^ (Table 1). All seven studies were rated intermediate quality, with attrition rates ranging from 18% to 84%; maternal methadone dose was variably reported; and where polydrug use was reported (five of seven studies), this ranged from 56% to more than 90% of mothers. The gestational age of participants was not stated in four studies.^38^, ^40^, ^41^, ^42^ Five studies reported rates of NAS between 67% and 92%; and no study described treatment for NAS. Compared with non‐exposed children, methadone exposure was associated with lower MDI, WMD of −4.43 (95% CI −7.24 to −1.63; Fig. 2a), and lower PDI, WMD of PDI −5.42 (95% CI −10.55 to −0.28; Fig. 2b).

Of the remaining 21 neurodevelopmental studies, 13 were rated as having intermediate quality and eight as poor quality. Infant Behavior Record was reported in two studies: Marcus et al. reported poorer motor performance at 4 months in 15 methadone‐exposed infants compared with 23 unexposed infants;^43^ and Wilson et al.^34^ matched 33 methadone‐exposed infants with 55 unexposed infants for maternal age, ethnicity, socio‐economic status, and marital status, and reported poorer fine motor coordination, less attentiveness, and lower motor scores on the Bayley Scales of Infant Development at 9 months of age, but no difference in cognitive scores. Schneider and Hans reported no difference in focused attention during free play at 24 months between 30 exposed and 44 unexposed toddlers.^44^

Suffet et al.^30^ reported MDI and PDI in the normal range at 1 year, with females performing better than males (MDI mean 108.8 vs 102.7, *p*<0.05; PDI mean 102.3 vs 95.7, *p*<0.05). This association persisted for cognition up to 2 years of age (MDI 99.2 vs 82.0, *p*<0.01). Bier et al.^45^ reported MDI scores in the normal range at 4 months of age, in a cohort of 165 methadone‐exposed infants, with no difference between those infants exposed prenatally to either low dose (<100mg/d) or high dose (≥100mg/d) methadone.

At 6 months of age, using the Griffiths Scales of Mental Development, McGlone et al.^46^ noted reduced median scores across all domains which persisted after adjustment for prenatal alcohol exposure and maternal smoking. This study included 81 methadone‐exposed infants and 26 non‐drug‐exposed infants, matched for gestation and socio‐economic status. Scores were also lower for infants who had been treated for NAS (median general quotient 95 vs 99, *p*<0.008). Bunikowski et al.^47^ reported reductions in quotients for two subscales (hearing and speech; intellectual performance) at 1 year of age in a case series of 18 prenatally exposed infants compared with 42 unexposed children.

Twelve studies evaluated children older than 2 years using a range of assessment tools (Table 2). Participants included 323 methadone‐exposed children and 321 unexposed children.

**Table 2:** Summary of 12 studies reporting neurodevelopmental outcomes in children beyond age 2y

Study	Quality rating^a^	Methadone‐exposed	Unexposed	Age^b^	Drug information^c^	Assessment tool	Main findings^d^ (results appear as methadone vs unexposed)
Strauss et al.^48^	B	31	27	5y	No information	MSCA	86.8 (13.3) vs 86.2 (16.2), ns
Lifschitz et al.^49^	B	26	41	3y 5mo	95% taking heroin or psychoactive drugs	MSCA	90.4 (13) vs 89.4 (10.8), ns
Rosen and Johnson^37^	B	39	21	3y	42 (mean of original cohort)	M‐P	44.6 (2.1) vs 46.3 (2.3) ns
Davis and Templer^53^	C	12	28	8y 6mo	No information	WISC‐R	89.58 (10.32) vs 96.32 (8.72) no *p*‐value
Wilson^41^	B	26	41	3–5y	No information	MSCA (GCI) Survey and school reports IQ testing	GCI: 90.4 (13.0) vs 89.4 (10.8) ns IQ 1–2 SD below norm 8% vs 5%, language disability 8% vs 5%, special education needs 16% vs 19%, behavioural problems 75% vs 48%, psychiatric referral 16% vs 5%
		12	12	6–11y			
Kaltenbach and Finnegan^38^	B	27	17	3y 6mo–4y 6mo	Mean dose 38.42	MSCA (GCI)	GCI: 106.5 (12.96) vs 106.05 (13.10), *t*=0.11
Sandberg et al.^54^	B	30	16	5–8y	39.5 (males), 38.7 (females) Original cohort, 68% polydrug use; 15% moderate to heavy alcohol intake	CGPQ^e^ CBAQ^e^ (males only)	Methadone‐exposed males showed more feminine game play than comparison males (*p*<0.04)
van Baar^39^	B	19	34	2y 6mo	No information	BSID (MDI, PDI, NDI) WWPA^e^	MDI 86 (15) vs 98 (16) *p*<0.05 PDI 102 (16) vs 100 (18) ns NDI 93 (16) vs 102 (22) WWPA 1.62 (1.03–2.66) vs 1.64 (1.28–2.52) ns
de Cubas and Field^50^	B	20	20	8y 6mo	No drug information. ‘Moderate alcohol use’	SBIS KABC‐A RATC CBCL^e^	SBIS: 97.6 vs 98.1, ns KABC‐A: 98.8 vs 102.4, no *p*‐value RATC: methadone‐exposed scored higher on anxiety, aggression, rejection, maladaptive outcome, *p*<0.01 for all CBCL: more behaviour problems *p*<0.05
van Baar and de Graaff^52^	B	23	32	3y 6mo	Polydrug use; 16 out of 35 heroin and cocaine, only 2 out of 35 solely methadone	SON‐IQ at 3y 6mo; RDLSC and RDLSE at 4y; RAKIT at 4y 6mo and 5y 6mo; IBR at 3y 6mo (*n*=22), 4y 6mo (*n*=23), 5y 6mo (*n*=22)	SON‐IQ: 99 (9) vs 109 (11), *p*<0.01 RDLSC: 46 (6) vs 52 (6), *p*<0.01 RDLSE: 46 (9) vs 50 (6), *p*<0.05 RAKIT 4y 6mo: 85 (11) vs 103 (15), *p*<0.01 RAKIT 5y 6mo: 90 (12) vs 102 (17), *p*<0.05 IBR: results median (range): 3y 6mo: free of fear: 9 (4–9) vs 6.5 (2–9), *p*<0.05; activity level: 6 (3–9) vs 5 (2–9), *p*<0.05; attention: 5 (1–7) vs 5.5 (1–9) *p*<0.05; fine motor: 3 (1–5) vs 3 (1–5), *p*<0.05 4y 6mo: cooperation: 6 (2–9) vs 7 (3–9), *p*<0.01; endurance: 4 (2–9) vs 6 (1–9), *p*<0.01; attention: 8 (2–9) vs 5 (2–8), ns 5y 6mo: cooperation: 6 (1–9) vs 8 (4–9), *p*<0.01; free of fear: 8 (2–9) vs 9 (5–9), ns; attention: 5 (2–8) vs 5 (3–9), ns
		26	32	4y			
		23	31	4y			
		22	30	6mo 5y 6mo			
Hunt et al.^51^	B	67	44	3y	No information	SBIS; VSMS; MSCA; RDLSC and RDLSE	SBIS: 99.9 (15.1) vs 107.5 (13.4), *p*<0.01 VSMSL: 38.4 (8.1) vs 46.1 (7.7), *p*<0.05 MSCA: 49.5 (8.7) vs 53.9 (8.3), *p*<0.05 RDLSC: 42.4 (11.6) vs 49.2 (11.4), *p*<0.05 RDLSE: 35.5 (7.9) vs 42.8 (12.8), *p*<0.05
Konijnenberg et al.^55^	B	24	0	4y	Mean 85.96^f^; polydrug use in 40% (illegal drug use), 25% alcohol	CBCL^e^	Scores >55 on aggressive behaviour and withdrawn behaviour

^a^Quality rating: A, good; B, intermediate; C, poor; based on modified Grading of Recommendations Assessment Development and Evaluation criteria (Table SI, online supporting information). ^b^Age expressed in months (mo) or years (y). ^c^Drug information includes mean daily methadone dose (in milligrams) and information on maternal polydrug use (defined as methadone plus any other drug use during pregnancy, excluding tobacco), where these are reported. Unless otherwise stated, all information in this column refers to methadone‐exposed group only. ^d^Scores are presented as mean values (standard deviation) unless otherwise stated. ^e^Questionnaire completed by parent or caregiver. ^f^Mean methadone dose excludes outlier daily dose of 660mg methadone. MSCA, McCarthy Scales of Childhood Abilities; ns, not significant; M‐P, Merril‐Palmer Scale; WISC‐R, Wechsler Intelligence Scale for Children–Revised; GCI, general cognitive index (used in the MSCA); SD, standard deviation; CGPQ, Child Game Participation Questionnaire; CBAQ, Child Behavior Attitude Questionnaire; BSID, Bayley Scales of Infant Development (original version 1969); MDI, mean developmental index (cognitive score); PDI, psychomotor developmental index (motor score); NDI, non‐verbal developmental index; WWPA, Werry‐Weiss Peters Activity Scale; SBIS, Stanford‐Binet Intellectual Scale; KABC‐A, Kaufman Assessment Battery for Children, achievement component (tests the acquired knowledge of fact); RATC, Robert's Apperception Test for Children (tests the child's perception of common interpersonal situations); CBCL, Child Behavior Checklist; SON‐IQ, Snijders‐Oomen Nonverbal Intelligence Test; RDLSC, Reynell Developmental Language Scales (Comprehensive); RDLSE, Reynell Developmental Language Scales (Expressive); RAKIT, Revision of the Amsterdam Children's Intelligence Test; IBR, Infant behaviour record; VSMS, Vineland Social Maturity Scale.

Six out of 10 studies measuring cognitive outcomes reported no difference between methadone‐exposed and unexposed children,^37^, ^38^, ^41^, ^48^, ^49^, ^50^ and four studies reported lower cognitive performance in methadone‐exposed children at 2 years 6 months,^39^ 3 years,^51^ 3 years 6 months,^52^ 4 years 6 months,^52^ 5 years 6 months,^52^ and 8 years 6 months^53^ respectively. The Reynell Developmental Language Scales were used in two studies (93 methadone‐exposed vs 76 unexposed children) at 3 years^51^ and at 4 years;^52^ both reported reduced performance in expressive and comprehensive language. Of the seven studies assessing behaviour, six reported more behavioural problems in methadone‐exposed children than in unexposed children.^41^, ^50^, ^51^, ^52^, ^54^, ^55^ Details of all studies reporting childhood neurodevelopmental outcome after prenatal methadone exposure are summarized in Table SII (online supporting information).

### Visual development and function

Twelve studies reported visual outcomes, five of which measured visual evoked potentials (VEPs; Table SIII, online supporting information). The VEP studies consisted of a total of 143 methadone‐exposed and 103 unexposed children, with one rated poor quality, three rated intermediate, and one rated good quality.

In two different cohorts, flash VEPs at 1 day to 4 days after birth were more frequently absent or immature and were smaller on average in methadone‐exposed infants compared with non‐exposed newborns.^56^, ^57^ At 4 months^58^ and at 6 months of age,^26^ pattern‐reversal and pattern‐onset VEP abnormalities persisted in methadone‐exposed infants. One follow‐up study of 10 3‐year‐old children previously tested at 4 months found no group difference in pattern‐reversal VEP peak times.^59^

A further six case series have described abnormal visual outcomes in a total of 108 methadone‐exposed children^60^, ^61^, ^62^, ^63^, ^64^, ^65^ (Table SIV, online supporting information). All six studies were rated as having poor quality evidence because they did not have comparison groups and did not correct for confounders owing to their observational design. However, collectively they describe common visual abnormalities, nystagmus (50 out of 108) and strabismus (51 out of 108), which may occur together (22 out of 108 cases). Nystagmus in all described cases was horizontal and either jerk or pendular in waveform.

In a case–control study of 100 methadone‐exposed infants, 81 of whom were followed up at 6 months of age, abnormal visual outcomes were present in 40% of methadone‐exposed children (nystagmus nine out of 81 cases, strabismus 20 out of 81 cases; both five out of 81) compared with two out of 26 non‐drug exposed infants matched for gestation and socio‐economic status.^26^ One intermediate quality study of methadone‐exposed 4‐year‐old children reported reduced visual selective attention in methadone‐exposed children compared with unexposed children.^66^

## Discussion

This systematic review of neurodevelopmental and visual outcomes of children born to opioid‐dependent mothers prescribed methadone in pregnancy has synthesized data from 41 studies (1441 children whose mothers were prescribed methadone and 842 children whose mothers were not prescribed methadone during pregnancy). In the meta‐analysis, we found that point estimates of MDI and PDI in children exposed to prenatal methadone compared with children whose mothers were not prescribed methadone are reduced at 6 months of age, and by 2 years the 95% CIs of these estimates make the possibility of no group difference in MDI and PDI unlikely. The emergence of difficulties as children grow older is well‐recognized after complications during the perinatal period and is likely to reflect the ontogeny of higher‐order functions through childhood. The finding of behavioural problems in six out of seven studies that measured this domain, and lower cognitive performance in four out of 10 studies that reported outcome after 2 years, suggests that children of opioid‐dependent mothers prescribed methadone may be at increased risk of longer‐term problems.

An association between prenatal methadone exposure and atypical visual development has been described, with significant differences in VEPs in infancy and childhood, reflecting altered visual pathways.^56^, ^58^ McGlone et al.,^26^ in their cohort of 81 methadone‐exposed and 29 unexposed infants at 6 months of age, describe a methadone‐attributable risk of abnormal visual assessment of 80%, after correcting for excess prenatal alcohol exposure. The prevalence of childhood strabismus and nystagmus in the methadone‐exposed population is higher than expected, which suggests that disorders of childhood visual function, as well as altered electrophysiological measures, are associated with prenatal methadone exposure.

Our findings are consistent with a recent meta‐analysis of five studies of infants and preschool children exposed to chronic intrauterine illicit heroin and/or prescribed methadone, which reported neurobehavioural impairment in the opioid‐exposed group.^67^, ^68^ Our data provide additional information by focusing on studies of women prescribed methadone, analysis of studies that reported a wide range of outcomes including visual development, and inclusion criteria designed to achieve maximum representation of the target population. Specifically, because use of prescribed and non‐prescribed drugs and tobacco is common among pregnant women prescribed methadone (but ascertainment and reporting of polydrug exposure in studies is variable),^69^ and because our purpose was to determine outcomes of methadone‐exposed children rather than to investigate causation, we took a pragmatic approach and did not attempt to exclude on the basis of polydrug use.

The data are also consistent with the observation that fractional anisotropy is reduced throughout the white matter skeleton of neonates born to mothers who were prescribed methadone,^15^ because neonatal fractional anisotropy is associated with later neurodevelopmental impairment.^70^, ^71^ More broadly, these results contribute to an emerging literature suggesting that exposure of the brain to psychoactive drugs during the perinatal period may modify its development.^72^, ^73^

A strength of this work is its pragmatic and systematic approach to summarizing childhood neurodevelopmental outcome after prescribed prenatal methadone exposure. We excluded studies of neonatal neurodevelopment to prevent confounding by NAS, and we excluded studies of alternative opioid substitutes to derive maximum inference about methadone.

However, limitations of included studies mean that the risk of impairment in children whose mothers were prescribed methadone may be biased. In particular, comparison groups were often poorly described beyond the definition of ‘non‐opioid exposed’, with inadequate control for socio‐economic status, or environmental factors, making it difficult to know who the methadone‐exposed children were being compared with. Reporting of maternal methadone dosing and polydrug or alcohol use was variable and therefore it was difficult to obtain an accurate exposure profile of included children; only one study examined prenatal exposure in all infants in detail using extensive toxicology.^57^ Finally, only 15 of the 41 studies were published in the past decade, which might affect application of results to contemporary populations because patterns of drug misuse change over time and strategies for MAT of opioid use disorder in pregnancy have evolved. For example, the dose of methadone prescribed for MAT in current practice is typically higher than that reported in historical studies. Further study of contemporary populations is required to determine the neurodevelopmental and visual outcomes of children born to opioid‐dependent mothers.

Improved understanding of the effects of prenatal opioid use disorder and its treatment, including the use of alternative substitutes, has been identified as a research priority.^74^ Buprenorphine has been evaluated as an opioid substitute in pregnancy. Although less severe NAS, improved growth, shorter hospital stay, and longer gestation are all reported in buprenorphine‐exposed compared with methadone‐exposed infants,^16^, ^75^, ^76^, ^77^ a recent Cochrane review concluded that there are insufficient data to establish whether buprenorphine is equivalent for all maternal outcomes, including adherence to treatment.^18^ Furthermore, confounding by indication could explain improved neonatal outcomes in buprenorphine groups.^17^ Therefore, there remains clinical equipoise about the safest opioid substitute for mother and child.

The data presented highlight that being born to an opioid‐dependent mother who has been prescribed maintenance methadone in pregnancy is associated with adverse visual and neurodevelopmental outcomes in infancy and early childhood, but deficiencies in the existing literature limit causal inference about harm and factors other than methadone per se could account for these observations. Further research into optimal management of opioid‐dependent pregnant women is required; future studies should consider fetal brain development and long term neurodevelopmental and visual outcomes of the child.

## Supporting information


**Table SI:** Quality assessment of 41 studies assessing childhood outcomes after prenatal methadone exposure

**Table SII:** Twenty‐nine studies reporting childhood neurodevelopmental outcomes after prenatal methadone exposure

**Table SIII:** Studies reporting childhood visual evoked potentials (VEPs) after prenatal methadone exposure

**Table SIV:** Studies reporting childhood visual outcomes after prenatal methadone exposure

**Figure S1:** Identification and selection. Preferred Reporting Items for Systematic Reviews and Meta‐Analyses (PRISMA) flowchart showing process of inclusion and exclusion of studies.
