# Supplementary material for: Childhood neurodevelopment after prescription of maintenance methadone for opioid dependency in pregnancy: a systematic review and meta‐analysis
Source: Dev Med Child Neurol. 2018 Dec 3;61(7):750–60. doi: 10.1111/dmcn.14117 (PMC6617808; doi:10.1111/dmcn.14117)
Supplement: Supplementary file 1 — Table SI: Quality assessment of 41 studies assessing childhood outcomes after prenatal methadone exposure [file DMCN-61-750-s002.doc]

| Supplemental Table 1. Quality assessment of 41 studies assessing childhood outcomes after prenatal methadone exposure | | | | | | | | | | |
| --- | --- | --- | --- | --- | --- | --- | --- | --- | --- | --- |
| First author and Year | Domain^a^ | Design ^b^ | Comparison unexposed group ^c^ | Population ^d^ and sample size ^e^ | Objective assessment tool ^f^ | Risk of Bias | | | Clear findings and statistical analysis ^j^ | Total quality score ^k^ |
|  |  |  |  |  |  | Blinded assessor ^g^ | Confounders ^h^ | Attrition ^i^ |  |  |
| Ramer 1975 | ND | 0 | 0 | 0 | 1 | 0 | 0 | 0 | 1 | 2 |
| Zarin-Ackerman 1976 | ND | 0 | 0 | 0 | 1 | 0 | 0 | 0 | 0 | 1 |
| Strauss 1976* | ND | 0 | 1 | ½ | 1 | 0 | 0 | 0 | 1 | 3 ½ |
| Kaltenbach 1979 ‡ | ND | 0 | 1 | 0 | 1 | 1 | ½ | 0 | 1 | 4 ½ |
| Strauss 1979 | ND | 0 | 1 | ½ | 1 | 1 | 0 | 0 | 1 | 4 ½ |
| Wilson 1981 | ND | 0 | 1 | ½ | 1 | 1 | ½ | 1 | 1 | 6 |
| Marcus 1982 | ND | 0 | 1 | 0 | 1 | 1 | ½ | 0 | 1 | 4 ½ |
| Chasnoff 1984 *‡ | ND | 0 | 1 | ½ | 1 | 0 | ½ | 0 | 1 | 4 |
| Suffet 1984 | ND | 0 | 0 | ½ | 1 | 0 | ½ | 0 | 0 | 2 ½ |
| Lifschitz 1985 | ND | 0 | 1 | ½ | 1 | 0 | 0 | 0 | 1 | 3 ½ |
| Rosen 1985* | ND | 0 | 1 | ½ | 1 | 0 | ½ | 0 | 1 | 4 |
| Kaltenbach 1986 | ND | 0 | 0 | 0 | 1 | 0 | 0 | 0 | 1 | 2 |
| Kaltenbach 1987 | ND | 0 | 1 | 1 | 1 | 0 | ½ | 0 | 1 | 4 ½ |
| Davis 1988 | ND | 0 | 1 | 0 | 1 | 0 | 0 | 0 | 1 | 3 |
| Doberczak 1988 | ND | 0 | 0 | 0 | 1 | 0 | 0 | 0 | 1 | 2 |
| Kaltenbach 1989 *‡ | ND | 0 | 1 | 0 | 1 | 0 | ½ | 1 | 1 | 4 ½ |
| Wilson 1989 ‡ | ND | 0 | 1 | 0 | 1 | 0 | ½ | 0 | 1 | 3 ½ |
| Sandberg 1990 | ND | 0 | 1 | ½ | 1 | 0 | 0 | 0 | 1 | 3 ½ |
| Van Baar 1990 *‡ | ND | 0 | 1 | ½ | 1 | 0 | ½ | 0 | 1 | 4 |
| De Cubas 1993 | ND | 0 | 1 | ½ | 1 | 0 | 0 | 0 | 1 | 3 ½ |
| Van Baar 1994 | ND | 0 | 1 | ½ | 1 | 0 | 0 | 0 | 1 | 3 ½ |
| Schneider 1996 | ND | 0 | 1 | ½ | 1 | 1 | 1 | 0 | 1 | 5 ½ |
| Bunikowski 1998 | ND | 0 | 1 | 0 | 1 | 0 | 1 | 0 | 0 | 3 |
| Hans 2001 ‡ | ND | 0 | 1 | ½ | 1 | 1 | ½ | 0 | 1 | 5 |
| Hunt 2008 | ND | 0 | 1 | ½ | 1 | 0 | ½ | 0 | 1 | 4 |
| Paul 2013 | ND | 0 | 0 | 0 | 1 | 0 | 0 | 0 | 1 | 2 |
| Konijnenberg 2015 | ND | 0 | 0 | ½ | 1 | 0 | 1 | 0 | 1 | 3 ½ |
| Bier 2015 | ND | 0 | 1 | ½ | 1 | 0 | 0 | 0 | 1 | 3 ½ |
| McGlone 2015 | ND | 0 | 0 | 1 | 1 | 0 | 1 | 1 | 1 | 5 |
| Nelson 1987 | V | 0 | 0 | 0 | 1 | 0 | 0 | 0 | 0 | 1 |
| Gaillard 2002 | V | 0 | 0 | 0 | 1 | 0 | 0 | 0 | 0 | 1 |
| McGlone 2008 | V | 0 | 1 | 1 | 1 | 1 | 0 | 0 | 1 | 5 |
| Hamilton 2010 | V | 0 | 0 | 0 | 1 | 0 | 0 | 0 | ½ | 1½ |
| Whitham 2010 | V | 0 | 1 | ½ | 1 | 1 | ½ | 0 | 1 | 5 |
| Gupta 2012 | V | 0 | 0 | ½ | 1 | 0 | 0 | 0 | 1 | 2 ½ |
| McGlone 2013 | V | 0 | 1 | 1 | 1 | 1 | 1 | 0 | 1 | 6 |
| Tinelli 2013 | V | 0 | 0 | 0 | 1 | 0 | 0 | 0 | 0 | 1 |
| McGlone 2014 | V | 0 | 1 | 1 | 1 | 1 | 1 | 1 | 1 | 7 |
| Konijnenberg 2015 | V | 0 | 1 | ½ | 1 | 0 | 0 | 0 | 1 | 3 ½ |
| Whitham 2015 | V | 0 | 1 | 0 | 1 | 0 | ½ | 0 | 1 | 3 ½ |
| Yoo 2017 | V | 0 | 0 | 0 | 1 | 0 | 0 | 0 | 1 | 2 |
| *indicates studies included in meta-analysis at 6 months, ‡indicates studies included in meta-analysis at 18–24 months.  ^a^ ND = neurodevelopmental, V = visual  ^b^ Randomised controlled trial = 1, observational study = 0  ^c^ Comparison unexposed group (not exposed to opioids) = 1, no comparison unexposed group = 0  ^d^ ≥36 w GA = ½, <36w GA = 0; ^e^ Sample size ≥20 = ½, sample size <20 = ½  ^f^ Objective assessment tool used = 1, no objective tool = 0  ^g^ Blinded = 1, not blinded = 0  ^h^ Confounder identified = ½, confounder adjusted for = ½, confounder not identified or not adjusted for = 0 (potential confounders included socio-economic status and/or polydrug use.)  ^i^ Attrition ≤20% of original cohort = 1, >20% of original cohort = 0, if not applicable due to study design, score 0  ^j^ Clear finding with statistical analysis = 1, findings not clear and no statistical analysis = 0  ^k^ Total scores 0–3 = poor, 3½ –6 = intermediate, 6½ –8 = good | | | | | | | | | | |
