# Supplementary material for: Childhood neurodevelopment after prescription of maintenance methadone for opioid dependency in pregnancy: a systematic review and meta‐analysis
Source: Dev Med Child Neurol. 2018 Dec 3;61(7):750–60. doi: 10.1111/dmcn.14117 (PMC6617808; doi:10.1111/dmcn.14117)
Supplement: Supplementary file 2 — Table SII: Twenty‐nine studies reporting childhood neurodevelopmental outcomes after prenatal methadone exposure [file DMCN-61-750-s003.doc]

| Supplemental Table 2. Twenty-nine studies reporting childhood neurodevelopmental outcomes after prenatal methadone exposure | | | | | | | | |
| --- | --- | --- | --- | --- | --- | --- | --- | --- |
| Study | Quality rating^a^ | Methadone-exposed | Unexposed | Age^b^ | Drug information^c^ | Assessment instrument | Main findings^d^  *appear as methadone vs unexposed* | Comments^e^ |
| Ramer *et al*, San Fransisco, 1975  Case series | C | 10  7  5  7  4 | 0  0  0  0  0 | 2-3 m  4-6 m  7-9 m  10-15 m  16-24 m | No dosing information  IUS (heroin metabolites in 6 urine) | BSID (MDI, PDI) at all ages | 2-3 m: MDI 117.3 (16.56); PDI 136.4 (18.67)  4-6 m: MDI 116.43 (17.91), PDI 116.29 (19.2)  7-9 m: MDI 110.4 (31.41), PDI 110.4 (30.26)  10-15 m: MDI 113.57 (13.25), PDI 105.71 (14.65)  16 -24m: MDI 99.25 (16.76), PDI 107.25 (18.7) | Original cohort 35 methadone-exposed infants (26 females, 9 males), no unexposed group.  One early neonatal death from meconium aspiration.  Mean GA at birth not stated; one infant was 36 weeks, the remainder were >36 weeks GA.  Pharmacological treatment for NAS in 14; Diazepam first line, paregoric (morphine) used as second agent in 2 cases, phenobarbital used for some cases of NAS. 10 infants received ‘medication for comfort’ and 4 ‘required regular sedative medications’. Medications used: diazepam, morphine and phenobarbital |
| Zarin-Acherman, New York, 1976  Case series | C | 16  14 | 0  0 | 3 m  6 m | Mean dose in 3^rd^ trimester 13.9; max 65;  No screening or PD information | BSID (MDI, PDI) at all ages | 3 m: MDI 101 (no sd); PDI 112 (no sd), *no statistics reported*  6 m: MDI 103 (no sd); PDI 107 (no sd), *no statistics reported* | Original cohort 42 methadone exposed infants, no unexposed group.  10 infants were preterm.  No information about NAS or treatment. |
| Strauss *et al*,* Baltimore, 1976  Prospective cohort study | B | 25  25  25 | 26  26  26 | 3 m  6 m *  1 yr | No information | BSID (MDI, PDI) at all ages | 3 m: MDI 112.5 (11.5) vs 115.3 (13.5); PDI 119.4 (9.1) vs 117.1 (14.5)  6 m*: MDI 115.7 (16.8) vs 114.3 (20.9); PDI 109.4 (12.2) vs 111.7 (14.5)  1 yr: MDI 113.4 (10.2) vs 114.8 (11.3); PDI 102.8 (11.0) vs 110.4 (9.8).  Decline in PDI between 3m and 12m in methadone exposed infants compared with unexposed: 16.6 points vs 6.7 points (p<0.01) | Original cohort 60 methadone-exposed infants and 53 unexposed infants; all infants were African-American.  Data reported only for infants who underwent BSID at all 3 ages. One case of sudden infant death in the methadone-exposed group. Matching of unexposed group not stated. GA at birth not stated. Assessor blinding not stated. No information about NAS or treatment |
| Kaltenbach *et al* ‡*,* Philadelphia, 1979  Prospective cohort study | B | 26  17 | 27  24 | 1 yr  2 yr ‡ | Mean dose for 1 yr cohort: 30; Mean dose for 2 yr cohort: 18  No screening or PD information | BSID (MDI) at all ages | 1 yr: MDI 103.4 (9.26) vs 109.4 (9.35), p<0.05  2 yr ‡: MDI 90.88 (8.26) vs 94.62 (11.93), ns | Original cohort 43 methadone-exposed infants and 51 unexposed (matched for maternal SES, ethnicity and medical conditions).  Assessors blinded to group. 1 year cohort had a mean GA of 40 weeks vs 39 weeks for unexposed infants. No range stated.  62% treated for NAS.  2 year cohort mean GA of 39 weeks vs 39 weeks, no range stated. 67% 2 year old cohort had been treated for NAS in the neonatal period. No pharmacological agent stated. Unclear whether overlap of participants assessed at 1 year and 2 years. |
| Strauss *et al,* Detroit, 1979  Prospective cohort study | B | 31 | 27 | 5 yr | No information | MSCA; Modified IBR (15 of 27 scales) | GCI 86.8 (13.3) vs 86.2 (16.2), ns  Modified IBR:  Gross bodily movement: 5.5 (1.4) vs 4.7 (1.4), p<0.05  Levels of energy: 3.4 (0.9) vs 2.8 (1.1), p<0.05  Fine motor co-ordination: 3.0 (0.8) vs 2.5 (1.0), ns  Irrelevant motor movement: 5.3 (1.7) vs 4.1 (1.7), p<0.01  Immaturity in interaction: 33% vs 7%, p<0.05 | Original cohort 60 methadone-exposed infants and 53 unexposed infants (matching not stated).  Same cohort as Strauss *et al* 1976 ^39^.  1 case of sudden infant death in the methadone-exposed group. All infants African American. Mean GA at birth not stated, range GA not stated. Assessor blinding not consistent. No information about NAS or treatment. |
| Wilson *et al,* Texas 1981  Prospective cohort study | B | 33 | 54 | 9 m | 20–60 (32 cases)  <20 (6 cases)  90 (1 case)  Maternal  urine screening;  PD >50%,  alcohol 10% Only 3/33 used solely methadone | BSID (MDI, PDI)  IBR | MDI 99.3 (15.5) vs 105.5 (15.6) ns; PDI 89.9 (12.6) vs 99.0 (14.5), p<0.01  IBR:  Poor fine motor co-ordination: 27/33 vs 27/55 unexposed (χ^2^ =8.80, p<0.01)  Less attentive: 8/33 vs 4/55 (χ^2^ =4.88, p<0.05) | Original cohort 39 methadone-exposed and 59 unexposed, one SIDS death in methadone-exposed group before assessment at 12 months.  Unexposed matched for maternal age, SES, marital status and ethnicity.  Mean GA not stated; 7/39 (18%) methadone-exposed group were preterm, compared with 6/57 (10%) of unexposed group. Assessor blinded to group. 34/39 severe NAS, treatment not stated |
| Marcus *et al,* Chicago, 1982  Prospective cohort study | B | 15 | 23 | 4 m | No  information | IBR | IBR sum score: 22.2 (2.57) vs 18.13 (2.72), p<0.001  Tension: 5.33 (0.9) vs 4.61 (1.47), ns  Activity: 4.93 (1.49) vs 3.87 (1.18), p<0.05  Interest in body motion: 5.4 (1.24) vs 3.96 (0.98), p<0.001  Co-ordination (gross motor): 3.13 (0.64) vs 2.68 (0.57), p<0.05  Co-ordination (fine motor): 3.4 (0.63) vs 2.96 (0.56), p<0.001 | No information about original cohort of infants.  All African-American infants. One case in methadone-group of SIDS and one stroke. Unexposed infants matched for maternal age and SES.  Mean GA not stated, range GA not stated. Assessors blinded to group. No methadone-exposed infants treated for NAS |
| Chasnoff *et al* *‡*,* Chicago, 1984  Prospective cohort study | B | 31  13  11  6 | 34  29  27  14 | 3 m  6 m *  1 yr  2 yr ‡ | Mean dose 14.6 ± 10.2  (range 5–40);  MUS;  4/31 used drugs in addition to heroin during pregnancy | BSID (MDI, PDI) at all ages | 3 m: MDI 105.0 (12.5) vs 99.2 (9.0); PDI 105.0 (18.6) vs 102.8 (7.0)  6 m*: MDI 105.9 (12.4) vs 111.0 (12.3); PDI 103.9 (9.0) vs 107.6 (15.1)  1 yr: MDI 104.2 (7.1) vs 105.8 (8.1); PDI 106.0 (14.4) vs 103.8 (12.5)  2 yr ‡: MDI 97.5 (16.1) vs 96.2 (15.9); PDI 99.3 (16.9) vs 98.2 (8.9)  *No p values stated* | Original cohort 39 methadone exposed and 34 unexposed. Unexposed group matched for maternal age, education, gravidity and smoking.  All infants were term (Ballard criteria), no range stated. Assessor blinding not stated. No information about NAS or treatment. |
| Suffet *et al,* New York, 1984  Case series | C | 131  106  99  35 | 0  0  0  0 | 3 m  6 m  1 yr  2 yr | Median 25–40  No screening information  No PD information stated, although a condition of joining the programme was detoxification from non-narcotics. | BSID (MDI, PDI) at all ages | 3 m: MDI 106.3 (13.5) PDI 112.1 (15.5)  6 m: MDI 108.1 (19.4) PDI 105.0 (14.5)  1 yr: MDI 97.7 (17.3) PDI 101.3 (16.1)  2 yr: MDI 89.3 (17.0) PDI 106.5 (19.1)  Compared by gender at all ages, girls vs boys:  1 yr: MDI 108.8 (11.2) vs 102.7 (12.8), p<0.05; PDI 102.3 (14.2) vs 95.7 (14.5), p<0.05  2 yr: MDI 99.2 (14.8) vs 82.0 (14.9), p<0.01  Significant associations between both BW and gender and MDI and PDI:   1. Positive correlation between birthweight and MDI at 6 months (p<0.001) PDI and 6 months (p<0.01), which is still present at 24 months: MDI p<0.01, PDI p<0.05). 2. Male vs female. 1 year: MDI 108.8 (11.2) vs 102.7 (12.8), p<0.05,   PDI 102.3 (14.2) vs 95.7 (14.5), p<0.05 | Original cohort 220 infants, all methadone exposed; 3.2% congenital anomalies; 5 deaths, 3 neonatal and 2 sudden infant deaths.  20% original cohort were <37 weeks GA at birth.  18.2% original cohort treated for NAS with paregoric (morphine). |
| Lifschitz *et al,* Houston, 1985  Prospective cohort study | B | 26 | 41 | Mean 3 yr 5 m  (range 3 yr – 5 yr 11 m) | No dosing or screening information;  95% PD use (heroin or psychoactive drugs) | MSCA (GCI) | GCI: 90.4 (13) vs 89.4 (10.8), ns  9 children (35%) in methadone-exposed group scored >1 sd below mean compared to 56% heroin exposed children and 22% unexposed children, p<0.01. | Original cohort 33 methadone-exposed and 57 unexposed infants (matched for maternal age, parity, SES and marital status).  Mean GA 38.8 weeks vs 39.2 weeks in unexposed group, range GA not stated. Assessors blinded to group. 88% methadone-exposed were treated for NAS, pharmacological agent not stated. |
| Rosen *et al* * ‡*,* New York, 1985  Prospective cohort study | B | 41  41  38  34  39 | 23  22  23  22  21 | 6 m *  1 yr  18 m  2 yr ‡  3 yr | 42 (mean of original cohort)  MUS;  PD use 56% of original cohort (BDZ, opiates, cocaine, barbiturates, TCA)15% reported “mod-severe” alcohol intake | BSID (MDI, PDI) at 6m,12m,18m and 2yr;  M-P at 3yr | 6 m *: MDI 95 (2.5) vs 100.7 (4.2), ns; PDI 101 (2.8) vs 105.1 (2.9), ns  1 yr: MDI 98.4 (2.7) vs 107 (2.8) p=0.05; PDI 94.9 (2.5) vs 102.8 (2.3) p=0.05  18 m: MDI 96 (2.3) vs 106.4 (3.6), p =0.05; PDI 92.6 (2.4) vs 105.3 (2.2) p=0.05  2 yr ‡: MDI 90.4 (2.6) vs 96.9 (3.1) ns; PDI 99.1 (2.7) vs 108 (2.7), p=0.05  3 yr: M-P 44.6 (2.1) vs 46.3 (2.3) ns.  *All scores are mean (SE)* | Original cohort 61 methadone-exposed infants and 32 unexposed infants (matched for maternal ethnicity, SES, infant gender, BW and GA).  15.4% preterm vs 11%, no range GA stated.  Assessor blinding not stated. 75% methadone-exposed had NAS; number treated pharmacologically not stated. |
| Kaltenbach *et al,* Philadelphia, 1986  Case series | C | 85  21  17  31  16 | 0  n/a  n/a  n/a  n/a | 6 months Group 1 Group 2  Group 3 Group 4 | No  information | BSID (MDI) | *MDI at 6 months reported by groups depending on the treatment for NAS:*  Group 1: paregoric (morphine) MDI 103 (no sd stated)  Group 2: phenobarbital MDI 104 (no sd stated)  Group 3: >1 agent MDI 103 (no sd stated)  Group 4: no treatment MDI 101 (no sd stated)  No difference between the 4 groups, p>0.1 | All infants were term. No mean GA stated.  69/85 treated for NAS with either paregoric (morphine), phenobarbital, or diazepam, or a combination. |
| Kaltenbach *et al,* Philadelphia 1987  Prospective cohort study | B | 105 | 63 | 6 months | 39  (5–85)  No screening or PD information | BSID (MDI) | 6 m: MDI 103.53 vs 104.39 (no sd stated) t=0.45, ns | Original cohort was 141 methadone exposed at birth. Unexposed group matched for maternal ethnicity, SES and medical background.  All infants >36 weeks GA, mean GA at birth 38.7 vs 39.4 weeks, no GA range stated.  Blinding of assessors not stated. 70% methadone-exposed were treated for NAS with paregoric (morphine) and/or phenobarbital. |
| Davis *et al,* California, 1988  Cross-sectional study | C | 12 | 28 | Mean  8.5yr vs 11.2 yr (range 6 – 15 yr) | No information | WISC-R | *Results stated are methadone subgroup vs unexposed at mean age of 8.5 yr vs 11.2 yr*  Verbal IQ: 89 (11.33) vs 94.29 (9.13) no p-value  Performance IQ: 92.75 (10.16) vs 100.0 (9.82) no p-value  Full scale IQ: 89.58 (10.32) vs 96.32 (8.72) no p-value | Original cohort 28 opiate-exposed, of which there was a subgroup of 12 methadone-exposed and 9 heroin exposed and 28 unexposed (no prenatal exposure to methadone but living in a ‘narcotic environment’ with an addicted parent/partner).  Mean GA not stated, range GA not stated.  Assessor not blinded. No information about NAS or treatment |
| Doberczak *et al,* New York, 1988  Cohort study | C | 8 vs 32  7 vs 16 | n/a  n/a | 5 – 7 m  8 – 16 m | Mean dose seizure: 56  Mean dose no seizure: 55.  MUS, IUS;  Both groups PD use with heroin, cocaine, barbiturates, AMP and BDZ. | BSID (MDI, PDI) at both ages | *Results stated are seizures vs no seizures*  5 - 7 m: MDI 103.1 (19.9) vs 111.5 (19.6), ns; PDI 114.7 (14.9) vs 103.4 (16.6), ns  8 - 16 m: MDI 114.0 (8.2) vs 109.7 (10.9), ns; PDI 109.7 (10.9) vs 99.5 (13.5), ns | Original cohort was 14 infants with methadone-related NAS-associated seizures and a comparison group of methadone-exposed infants with no seizures. No unexposed comparison group.  Mean GA of seizure group 39 weeks (range 33 – 43 weeks).  Mean GA of no seizure group 39 weeks (range 33 – 42 weeks). Assessor blinded to infant history. Treatment for NAS with morphine and/or phenobarbital. |
| Kaltenbach *et al**‡*,* Philadelphia, 1989  Prospective cohort study | B | 27  27  27  27 | 17  17  17  17 | 6 m *  1 yr  2 yr ‡  3.5 – 4.5 yr | Mean dose 38.42;  No screening or PD information | BSID (MDI) at 6m, 1 yr and 2 yr.  MSCA (GCI) at 3.5-4.5 yr | 6 m*: MDI 107.9 (12.23) vs 105.6 (7.31) no p value  1 yr: MDI 102.5 (11.38) vs 106 .53 (6.41), no p value  2 yr ‡: MDI 100.9 (18.04) vs 103.9 (11.49) no p value  3.5 - 4.5 yr: GCI 106.5 (12.96) vs 106.05 (13.10), *t*=0.11 | Limited information about original cohort. Unexposed group were matched for maternal ethnicity and SES. GA infants not stated, no GA range stated. Blinding of assessors not stated. 92% were treated for NAS, pharmacological agent not stated. |
| Wilson *et al,* Texas ‡, 1989  Prospective cohort study | B | 33  29  42  26  12 | 54  42  48  41  12 | 9 m  18 m  2 yr ‡  3 – 5 yr  6 – 11 yr | No dosing information  MUS;  93% used psychoactive drugs | BSID (MDI) at 9m, 18m, 2yr.  MSCA (GCI) at 3-5yr;  School performance 6-11 yr (survey, school reports and IQ testing) | 9 m: published in Wilson *et al* ^12^ 1981  18 m: MDI 92 (14.5) vs 97.4 (14.4) ns  2 yr ‡: MDI 88.8 (15.5) vs 90.2 (14.6) ns  3 – 5 yr: GCI 90.4 (13.0) vs 89.4 (10.8) ns  6 – 11 yrs: IQ 1- 2 sd below norm 8% vs 5%; Language disability 8% vs 5%; Special education needs 16% vs 19%; Behavioural problems 75% vs 48%; Psychiatric referral 16% vs 5%. | Original cohort 39 methadone-exposed, 57 unexposed infants (matched for maternal age, ethnicity, SES and marital status).  Mean GA not stated for either group, range GA not stated.  Assessor blinding not stated.87% original cohort treated for NAS, pharmacological agent not stated. |
| Sandberg *et al,* New York, 1990  Prospective cohort study | B | 30 | 16 | 5 – 8 years | 39.5 (boys), 38.7 (girls)  MUS;  Original cohort, 68% PD use; 15% moderate to heavy alcohol intake. | CGPQ^f^  CBAQ^f^ (boys only) | Methadone-exposed boys showed more feminine game play than unexposed boys (p<0.04). No significant differences in girls. Overall CBAQ scores were not significantly different between methadone-exposed and unexposed boys.  Group split by gender and by PD use creating: boys methadone only (n=5) and boys methadone + PD (n=9)  In the methadone only boys group (n=5), ANCOVA with age as covariate of individual elements of the feminine behaviour sub-scale showed higher (more feminine behaviour) scores for 2 items:  “he is good at imitating females”, p<0.001, “he dresses in female clothing”, p<0.05  In the PD boys group (n=9) there were higher feminine scores on “he is good at imitating females”, p<0.05, and “he does things with female relatives’, p<0.05 | Original cohort 61 methadone-exposed infants and 32 unexposed infants (matched for maternal ethnicity, SES, infant gender, BW (±250g), GA (±2w) and APGAR score).  2 methadone-exposed infants from original cohort died of sudden infant death.  GA at birth and range GA not stated. NAS treatment not stated.  Mother or primary care-giver completed questionnaire. |
| Van Baar *et al ** ‡*,* Netherlands, 1990  Prospective cohort study | B | 21  21  18  21  19 | 37  34  34  34  34 | 6 m *  1 yr  18 m  2 yr ‡  2.5 yr | No dosing or screening information;  Original cohort; 6 IV drug users;  94% used multiple drugs; 60% use cocaine | BSID (MDI, PDI, NDI) at all ages  WWPA^f^ at  18 m (n=14),  2 yr (n=16) and 2.5 yr (n=15) | *6 m MDI 103 (12) vs 107 (13); PDI 116 (18) vs 114 (21); NDI 105 (13) vs 109 (14)  1 yr: MDI 108(13) vs 114 (17); PDI 112 (21) vs 119 (20); NDI 109 (12) vs 112 (18)  18 m: MDI 94 (14) vs 99 (19); PDI 108 (20) vs 112 (19); NDI 97 (15) vs 99 (17)  2 yr ‡: MDI 86 (15) vs 98 (16) p<0.05; PDI 102 (16) vs 100 (18) ns; NDI 93 (16) vs 102 (22)  2.5 yr: MDI 87 (15) vs 101 (20) p<0.05; PDI 96 (19) vs 101(24) ns; NDI 100 (19) vs 108 (19) ns  *WWPA^d^, medians (range)*  18m: 1.78 (1.40 – 2.62) vs 1.73 (1.23 – 2.52) ns  2y: 1.66 (1.33 – 2.660 vs 1.76 (1.19 – 2.52) ns  2.5 yr: 1.62 (1.03 – 2.66) vs 1.64 (1.28 – 2.52) ns;  Methadone-exposed had lower MDI at 2 and 2 ½ years due to delayed early language. No differences in motor development, non-verbal development or hyperactivity scores. | Original cohort 35 methadone-exposed and 37 unexposed infants (not matched).  In methadone-exposed group 9/35 were preterm but results presented are subgroup of term only methadone-exposed infants.  Assessor blinding not stated. 28/35 (80%) were treated for NAS, pharmacological agent not stated |
| De Cubas *et al,* Miami, 1993  Cross-sectional study | B | 20 | 20 | Mean age 8.5 vs 7.8 yr | No drug information;  “moderate alcohol use” | SBIS;  KABC-A;  RATC;  CBCL^f^ | SBIS: 97.6 vs 98.1 ns.  Within methadone-exposed group: NAS (n=5) vs No NAS (n=15) 89.9 vs 100.2, *t* =3.65, *p*<0.002  KABC-A: 98.8 vs 102.4, *no p value*; Faces and places subtest 95 vs 103, p<0.02;  RATC: Methadone-exposed scored higher on anxiety, aggression, rejection and maladaptive outcome, p<0.01 for all;  CBCL: More behaviour problems [depressed, social withdrawal, somatic complaints, hyperactive, aggressive, delinquent, internalising and externalising behaviour] reported by parents in methadone group, p<0.05 all categories. | Unexposed group matched for demographics (not stated) age/grade level, sex, ethnicity, SES, family structure, maternal education, maternal alcohol/tobacco, perinatal complications.  Mean GA not stated; In methadone-exposed one child was preterm; In the unexposed group 10/20 had “perinatal complications such as prematurity or SGA”.  Assessor blinding not stated. 3/20 treated for NAS with phenobarbital |
| Van Baar *et al,* Netherlands, 1994  Prospective cohort study | B | 23  26  23  22 | 32  32  31  30 | 3.5 yr  4 yr  4.5 yr  5.5 yr | No dosing information;  Maternal interview and MUS, IUS;  PD use: 16/35 heroin and cocaine, 2/35 solely methadone | SON IQ at 3.5 yr;  RC and RE at 4 yr;  RAKIT at 4.5 and 5.5 yr; IBR at  3.5 yr (n=22),  4.5 yr (n=23)  5.5 yr (n=22) | SON IQ 99 (9) vs 109 (11), p<0.01  RC 46 (6) vs 52 (6), p<0.01  RE 46 (9) vs 50 (6), p<0.05  RAKIT 4.5 yr: 85 (11) vs 103 (15), p<0.01; 14 / 23 methadone-exposed children had developmental delay (defined as scores >1 sd), p<0.01  RAKIT 5.5yr: 90 (12) vs 102 (17), p<0.05  IBR: *Results median(range)*  3.5 yr: Free of fear: 9 (4-9) vs 6.5 (2-9), p<0.05; Activity level: 6 (3-9) vs 5 (2-9), p<0.05; Attention: 5 (1-7) vs 5.5 (1-9) p<0.05; Fine motor: 3 (1-5) vs 3 (1-5), p<0.05  4.5 yr: Co-operation: 6 (2-9) vs 7 (3-9), p<0.01; Endurance: 4 (2-9) vs 6 (1-9), p<0.01; Attention: 8 (2-9) vs 5 (2-8), ns  5.5 yr: Co-operation: 6 (1-9) vs 8 (4-9), p<0.01; Free of fear: 8 (2-9) vs 9 (5-9), ns; Attention: 5 (2-8) vs 5 (3-9), ns.  After correcting for behaviour that differed between groups, significant differences between methadone-exposed and unexposed children still existed at 3.5 years for SON IQ, p<0.05, and at 4.5 years for RAKIT, p<0.01, but not at 5.5 years for RAKIT, p=0.13 | Original cohort 35 methadone-exposed vs 35 unexposed (unmatched).  Mean GA for original cohort 38 weeks vs 39.7 weeks, no range stated; 7/23 methadone exposed infants were preterm, range of GA not stated.  Blinding of assessors not stated.  28/35 were treated for NAS, all treated with phenobarbital. |
| Schneider *et al,* Chicago, 1996  Prospective cohort Study | B | 30 | 44 | 2 yr | Mean <20  (range 3 – 40)  *“most women occasionally used cannabis, alcohol and BDZ”* | Focus Ratio | Focus ratio at 2 yr: 0.35 (0.15) vs 0.31 (0.13) ns  ANCOVA to unexposed for cannabis (F(1,69 = 0.16), nicotine (F(1,69) = 0.16) and alcohol (F(1,69 = 0.04) ns | Unexposed comparable for low income. 2 methadone-exposed were preterm (30w and 33w).  Attrition: 4 children died before 2 years of age in the methadone-exposed group, and one had massive cerebral haemorrhage. 1 in unexposed group withdrew following diagnosis of cerebral palsy. Assessors scoring video-taped encounter were blinded to group.  No methadone-exposed child was treated for NAS. |
| Bunikowski *et al,* Germany, 1998  Prospective cohort study | C | 18 | 42 | 1 yr | No  information | Griffiths subscales: | *Methadone sub group data extracted:*  Hearing and speech: 99.7 (8.1) vs 98.8 (9.1)  Intellectual performance at 1 yr: 104.3 (11.6) vs 108.5 (11.1)  *No p values as these results taken out of text as subgroup analysis* | Original cohort 46 opiate exposed versus 47 unexposed infants (matched for maternal smoking). 27 opiate exposed underwent Griffiths assessment at 1 year; opiate group split into methadone (n=18) and heroin (n=9), sub-group analysis reported  Mean GA not stated; 13/34 opiate exposed were preterm, range not stated  Assessor blinding not stated. 28/46 treated for NAS with phenobarbital |
| Hans *et al,* Chicago ‡, 2001  Prospective cohort study | B | 33  33  33  33  33 | 45  45  45  45  45 | 4 m  8 m  1 yr  18 m  2 yr ‡ | Mean <20  (Range 3 - 40mg);  Maternal interview and MUS;  PD reported: 13/33 cocaine,  18/33 cannabis,  9/33 mild-mod alcohol use,  2/33 heavy alcohol use | BSID (MDI, PDI) at all ages | 4 m: MDI 111 (12.3) vs 114 (15.1); PDI 116 (12.5) vs 121 (12.3)  8 m: MDI 116 (19.5) vs 120 (20.2); PDI 111 (12.4) vs 111 (12.4)  1 yr: MDI 107 (14.3) vs 109 (13.7); PDI 106 (18) vs 110 (17.7)  18 m: MDI 95 (16.3) vs 103 (13.1); PDI 105 (14.2) vs 109 (14.9)  2 yr ‡: MDI 92 (12.7) vs 96 (12.3); PDI 100 (14.2) vs 108 (14.9)  Mean across all ages:  MDI 104 (7.8) vs 108 (8.3), p<0.05; PDI 108 (9.2) vs 112 (10.4), ns | Original cohort 47 methadone-exposed infants and 45 unexposed infants (matched for maternal age, SES and IQ).  4 sudden infant deaths in the methadone-exposed group. All infants included were African-American.  3 subjects not tested at 2 years, not stated which group these subjects were in. Scores were estimated, based on their median 18 month scores and the average scores for the entire sample.  Mean GA not stated, no GA range stated. Assessors blinded to group. No information about NAS or treatment |
| Hunt *et al*, Australia, 2008  Prospective cohort study | B | 79  67 | 61  44 | 18 m  3 y | No  information | BSID III (MDI, PDI) at 18 m;  VL at 18m and 3 yr;  SBIS and MSCA (GCI) at 3 yr;  RC and RE at 3 yr | 18 m: MDI 88.2 (16.4) vs 105.02 (23), p <0.001; PDI 107.5 (16.8) vs 110.13 (14.7)  SBIS: 99.9 (15.1) vs 107.5 (13.4), p<0.01  GCI: 49.5 (8.7) vs 53.9 (8.3), p<0.05  VL 18 m: 113.2 (15.6) vs 119.15 (17.5), p<0.05  VL 3 yr: 38.4 (8.1) vs 46.1 (7.7), p<0.05  RC 42.4 (11.6) vs 49.2 (11.4), p<0.05; RE 35.5 (7.9) vs 42.8 (12.8), p<0.05 | Original cohort 133 methadone exposed and 103 unexposed infants (matched for maternal age, height and ethnicity).  In families lost to follow up, 10/133 methadone taking mothers had died before their child was 3 years old.  Original cohort mean GA at birth 37.7 vs 40.2 weeks, 32/133 were preterm, all singleton pregnancies.  Assessor blinding not stated.  74 /133 infants were treated for NAS with morphine. No NAS related seizures. |
| Paul *et al*, Maine, 2013  Case series | C | 19  17  24 | 0  0  0 | 4-15 d  16 – 32 d  33 – 120 d | No drug information;  Maternal questionnaire asked about alcohol use | Auditory ERP:  P2 amplitude; P2 latency; Mismatch negativity | P2 amplitude greater in 16 – 32 d and 22 – 120 d in the frontal region, p<0.001 both  Latency to oddball stimulus longer only in in 4 – 15 day group, p 0.04, but not in the  Mismatch negativity amplitude became less negative as P2 amplitude to oddball stimulus increased at Fz, r^2^ 0.38, p<0.001 | Three separate cohorts of infants, no unexposed group.  Mean GA at birth for all groups was 38 weeks, no range stated.  All infants in the 4 – 15 day group were receiving pharmacological treatment for NAS at the time of ERP testing. Pharmacological agent used for NAS treatment not stated. |
| Konijnenberg *et al*, Oslo, 2015  Prospective cohort study | B | 24 | 0 | Mean 52 m | Mean 85.96^g^  Maternal interview and medical records, IUS;  PD reported in 40% and  25% alcohol | CBCL^f^ | Methadone-exposed children aged 4 scored >55 on aggressive behaviour and withdrawn behaviour. | Part of a larger cohort study comparing buprenorphine with methadone.  No unexposed group. Mean GA at birth 38.7 weeks. Inclusion of preterm infants not stated. No assessor blinding.  13/24 treated for NAS, pharmacological agent not stated |
| Bier *et al*, Boston, 2015  Retrospective cohort | B | High 81  Low 84 | n/a  n/a | 4 m | Mean dose not stated.  IUS;  Co-treatment with psychiatric medication in 43% (high dose) and 10% (low dose) | BSID III (MDI);  AIMS | *Subgroup methadone data extracted from paper. Scores presented are low dose vs high dose*  MDI 96.6 (7) vs 94.3 (9), ns  AIMS percentile (sd): 44.8 (24) vs 38.1 (24) ns  High dose methadone associated with decreased HC z-score compared with low dose methadone, p<0.025.  *No statistics reported on BSID or AIMS scores comparing low vs high, as subgroup data extracted from paper*  Regression analysis confirmed an association between high dose methadone and lower HC z-score, p <0.025 | Part of a study comparing low doses of methadone, high doses of methadone and buprenorphine. No unexposed comparator group.  Subgroup results presented.  Mean GA low group 37 weeks, 18/84 preterm.  Mean GA high group 38 weeks, 13/81 preterm.  Assessor blinded to infant background  NAS treated in 72/84 in low group and 73/81 in the high group, treated with morphine and /or phenobarbital |
| McGlone *et al,* UK, 2015  Prospective cohort study | B | 81 | 26 | 6 m | No dosing information  MUS, IUS;  Infant meconium;  75% opiates  67% BDZ  64% cannabis  26% stimulants | Griffiths Scales of Mental Development 1996 revision. | *All data median (IQR) and adjusted p values (correcting for maternal smoking and alcohol)*  GQ: 97 (93 -100) vs 105 (101-108) p<0.001;  Locomotor: 102 (97 – 107) vs 111 (101-111) p=0.006;  Personal-social: 94 (88-96) vs 99 (94 – 103) p=0.001;  Language – hearing: 105 (105 – 109) vs 109 (105 – 109) p=0.007;  Eye – Hand: 94 (86 – 99) vs 104 (99 – 104) p=0.001;  Performance: 96 (86-100) vs 101 (101 – 111) p=0.002.  Adjusted p values correct for maternal smoking and excess alcohol.  8/81 methadone-exposed had GQ <85. All infants in unexposed group scored ≥95.  Visual impairment independently associated with adverse neurodevelopmental outcome defined as GQ <85 (P<0.001). Infants treated for NAS vs those not treated for NAS had different scores: Median GQ 95 vs 99, (p=0.008). Infants exposed to multiple drugs had lower scores for locomotor and hand-eye skills (p=0.002) | Original cohort 100 methadone-exposed infants and 50 unexposed infants  (matched for gestation, birthweight and maternal postcode).  Infants excluded if GA <36 weeks. Single assessor, not blinded to allocation.  39 infants treated for NAS with morphine, 15/39 treated with morphine and phenobarbital. |
| *indicates studies included in meta-analysis at 6 months, ‡ indicates studies included in meta-analysis at 2 years.  ^a^ Quality rating: A = good, B = intermediate, C = poor, based on modified GRADE criteria (Supplemental Table 1); ^b^ Age expressed in days (d), months (m) or years (yr);  ^c^ Drug information includes mean daily methadone dose (in milligrams), maternal urine screening (MUS) and/or infant urine screening (IUS) for drug exposure and information on maternal polydrug (PD) use, where these are reported. Unless otherwise stated, all information in this column refers to methadone-exposed group only; ^d^ Scores are presented as mean values (standard deviation) unless otherwise stated; ^e^ Comments include information on attrition, matching, gestation, blinding, proportion of infants treated for NAS and pharmacological treatment for NAS, where this is provided in the original publication; ^f^ Questionnaire completed by parent or care-giver; ^g^ Mean methadone dose excludes outlier daily dose of 660mg methadone.  AIMS = Alberta Infant Motor Scales, ANCOVA = analysis of co-variance, BDZ = benzodiazepine, BSID = Bayley Scales of Infant Development, 1969, BSID III = Bayley Scales of Infant Development, 3^rd^ edition, BW = birth weight, CBAQ = Child Behaviour Attitude Questionnaire CBCL = Child Behaviour Checklist, GCI = Cognitive General Index (used in the MSCA), CGPQ = Child Game Participation Questionnaire, ERP = event-related-potentials, Focus ratio = focussed attention : total play time (as observed by an assessor during 3 minutes of free play), GA = gestational age, GQ = Griffiths Quotient, KABC-A = Kaufman Assessment Battery for Children, achievement component (tests the acquired knowledge of fact), NAS = neonatal abstinence syndrome, NDI = Non-verbal Developmental Index, PD = polydrug (defined as methadone plus any other drug use during pregnancy, excluding tobacco), RATC = Robert’s Apperception Test for Children (tests the child’s perception of common interpersonal situations), RAKIT = Revision of the Amsterdam Children’s Intelligence Test, RC = Reynell Developmental Language Scales, comprehension, RE = Reynell Developmental Language Scales, expression, SBIS = Stanford-Binet Intellectual Scale, sd = standard deviation, SES = socio-economic status, SIDS = sudden infant death syndrome, SGA = small for gestational age, SON-IQ = Snijders-Oomen Nonverbal Intelligence Test, Vineland SM = Vineland social maturity Scale, WICS-R = Weschler Intelligence Scale for Children – Revised, WWPA = Werry-Weiss Peters Activity Scale | | | | | | | | |
