# Supplementary material for: Childhood neurodevelopment after prescription of maintenance methadone for opioid dependency in pregnancy: a systematic review and meta‐analysis
Source: Dev Med Child Neurol. 2018 Dec 3;61(7):750–60. doi: 10.1111/dmcn.14117 (PMC6617808; doi:10.1111/dmcn.14117)
Supplement: Supplementary file 3 — Table SIII: Studies reporting childhood visual evoked potentials (VEPs) after prenatal methadone exposure [file DMCN-61-750-s004.docx]

**Table SIII:** Studies reporting childhood visual evoked potentials (VEPs) after prenatal methadone exposure

| Study | Quality rating^a^ | Methadone-exposed | Unexposed | Age^b^ | Drug information^c^ | Visual assessment | Main findings^d^ (results appear as methadone vs unexposed) | Comments^e^ |
| --- | --- | --- | --- | --- | --- | --- | --- | --- |
| McGlone et al.^57^ | B | 21 | 20 | 1d–4d | No methadone information. ‘majority used BDZ’  IUS; BDZ 8 out of 13, cocaine 2 out of 13, cannabinoids 1 out of 13. | Flash VEP^f^  Amplitude  Peak latency P2 and N3 | Days 1–4, methadone-exposed infants had fewer typical VEPs, more immature waveforms, and non-detectable VEPs in 5 out of 21 cases, *p*<0.01  Median amplitude 10.6mV vs 24.4mV, *p*<0.001.  After 1wk, 14 out of 21 methadone-exposed infants had repeat VEPs: remained low amplitude, median 11.3mV  Peak latencies for P1 and N3 did not differ significantly between groups. 24% methadone-exposed had no detectable VEP but all unexposed infants had a detectable VEP | Unmatched unexposed comparison group.  All infants ≥37wks gestational age.  VEP assessors blinded to group.  VEP measured at 7d if methadone-exposed infant still in hospital.  7 out of 21 infants treated for NAS with morphine. |
| Whitham et al.^59^ | B | 22 | 33 | 4mo | Mean methadone 45.4 (range 15–100)  MUS; 77% positive in methadone mothers,  33% positive control mothers (opioids=3, cannabis=8, BDZ=2) | Pattern-reversal VEP (P1 latency)  Binocular viewing | P1 latencies (ms) to 48min 136.25 (18.02) vs 124.34 (12.35)–12ms (95% CI 4–20ms, *p*=0.0052)  P1 latencies (ms) to 69min 134.99 (33.46) vs 119.92 (11.74)–15ms (95% CI 2.4–28ms, *p*=0.021)  Multiple regression analysis revealed that prenatal methadone exposure is a significant predictor of prolonged P1 latency in response to checks of 48’ retinal arc (regression coefficient −0.30, *p*<0.05) after correcting for confounders | Part of a buprenorphine vs methadone vs unexposed study. Original cohort 72 opioid mothers and 35 mothers not taking opioids (unexposed group matched for maternal age, parity, gravida, self-reported alcohol use, and smoking).  Mean gestational age at birth 38.09wks vs 38.85wks, no range gestational age.  VEP assessors were blinded. 11 out of 22 treated for NAS with morphine. |
| McGlone et al.^58^ | B | 100 | 50 | 1–3d | Maternal history (*n*=100), MUS (*n*=84)  IUS (*n*=70), and meconium, *n*=74.  30 comparison infants had meconium samples | Flash VEP^f^: when present, amplitude and implicit times of peaks and troughs measured (P1, P2, N3, P3) | Methadone-exposed were less likely to demonstrate P1 components of VEP (21% vs 48%, *p*=0.001) and N2 components of the VEP (38% vs 60%, *p*=0.011)  Methadone-exposed infants had smaller amplitude VEPs median 27μV vs 39μV, *p*<0.001 and had more immature or atypical VEPS, *p*=0.001. All differences persisted after correcting for confounders (head circumference, cigarette smoking, excess alcohol)  No association between maternal methadone dose and VEP abnormalities.  NAS did not impact on VEP amplitude, morphology or implicit times | Unexposed group matched for gestational age (completed weeks), bodyweight (±250g), Carstairs deprivation index (±1)  Excluded infants <36wk gestational age. Two VEP assessors, one blinded to group.  All VEPs recorded before any NAS treatment. 48 out of 100 treated for NAS with morphine, 22 out of 48 required two drugs (morphine and phenobarbital) |
| McGlone et al.^47^ | A | 81^g^ | 26^g^ | 6mo | No methadone dosing information  PD use in 90%: 75% opioids; 67% BDZ; 64% cannabis; 26% stimulants.  20 out of 46 tested for FFAE had elevated levels.  3 out of 18 control infants had elevated FFAE and two tested positive for cannabinoids | Pattern-onset VEP: present or absent.  C2 latency  C2 amplitude | C2 latency (ms) at 120min 115 vs 99, *p*=0.019, at 60min 115 vs 106, *p*=0.036, at 15’ 128 vs 108, *p*=0.0002  C2 amplitude (μV) at 120min 24 vs 26, *p*=0.091, at 60min 24 vs 34, *p*=0.003, at 15min 10 vs 17, *p*=0.003  Methadone-exposed infants significantly less likely to have a VEP in response to the smallest check size of 15min (51 out of 70 vs 24 out of 24, *p*=0.006). Overall, 70% of methadone-exposed infants had one or more abnormal VEP parameter | Initial cohort 100 methadone exposed vs 50 unexposed (follow-up study of same cohort as McGlone et al.^58,70^)  Infants <36wk gestational age excluded. One paediatrician and one optometrist assessed vision. Optometrist blinded to group. Corrected for confounding effect of excess prenatal alcohol exposure  55 out of 81 treated NAS with morphine. No infant had a clinical diagnosis of fetal alcohol syndrome |
| Whitham et al.^60^ | B | 10^h^ | 15^h^ | 3y | No methadone information  PD use 5 out of 10 BDZ  7 out of 10 positive urine screen | Binocular pattern reverse VEP latencies | P100 latencies: 48min retinal arc: 103.7 vs 104.7, *p*=0.47; 69min retinal arc: 101.1 vs 102.7, *p*=0.65  Multiple regression analysis showed that head circumference at time of testing was a significant predictor of P100 latency for checks of 69min, *p*=0.04 | Follow-up study of Whitham et al.^59^  Inclusion of infants born preterm not known as gestational age not stated.  11 methadone-exposed infants tested; one had significantly prolonged P100 latencies, and was excluded from the analysis as an ‘outlier’  Blinding of assessor not stated. 6 out of 10 treated for NAS |

^a^Quality rating: A, good; B, intermediate; C, poor; based on modified Grading of Recommendations Assessment Development and Evaluation criteria (Table SI, online supporting information). ^b^Age expressed in days (d), months (mo), or years (y). ^c^Drug information includes mean daily methadone dose (in milligrams), maternal urine screening, and/or infant urine screening for drug exposure and information on maternal polydrug use (defined as methadone plus any other drug use during pregnancy; excluding tobacco), where these are reported. Unless otherwise stated, all information in this column refers to methadone-exposed group only. ^d^Scores are presented as mean values (standard deviation) unless otherwise stated; ^e^Comments include information on attrition, matching, gestation, blinding, proportion of infants treated for neonatal abstinence syndrome (NAS), where provided in the original study. ^f^Flash visual evoked potential categorized as typical, atypical, immature, or not detectable; ^g^Same cohort as McGlone et al.,^58,70^ examined at 6mo. ^h^Same cohort as Whitham et al.,^59^ examined at 3y. BDZ, benzodiazepine; IUS, infant urine screening; NAS, neonatal abstinence syndrome; MUS, maternal urine screening; FFAE, free fatty acyl esters (a biomarker of alcohol consumption in utero).
