# Supplementary material for: Childhood neurodevelopment after prescription of maintenance methadone for opioid dependency in pregnancy: a systematic review and meta‐analysis
Source: Dev Med Child Neurol. 2018 Dec 3;61(7):750–60. doi: 10.1111/dmcn.14117 (PMC6617808; doi:10.1111/dmcn.14117)
Supplement: Supplementary file 4 — Table SIV: Studies reporting childhood visual outcomes after prenatal methadone exposure [file DMCN-61-750-s005.docx]

**Table SIV:** Studies reporting childhood visual outcomes after prenatal methadone exposure

| Study | Quality rating^a^ | Methadone-exposed | Unexposed | Age^b^ | Drug information^c^ | Visual assessment | Main findings^d^ (results appear as methadone vs unexposed) | Comments^e^ |
| --- | --- | --- | --- | --- | --- | --- | --- | --- |
| Nelson et al.^61^ | C | 29 | 0 | 6mo–27mo | Mean 40.8  MUS; polydrug use reported | Strabismus | 7 out of 29 had strabismus. Four with esotropia diagnosed at a mean age of 10.5mo, three with exotropia diagnosed at a mean age of 15mo  Paper compared the strabismus group with the non-strabismus group to look for associations | Original cohort of 40 infants examined in neonatal period. No unexposed comparison group.  2 out of 29 infants were born preterm. 21 out of 29 treated for NAS, drug not stated |
| Gaillard et al.^62^ | C | 5 | n/a | 13mo  30mo  4mo  4mo  2mo | Case 1: BDZ, cannabis  Case 2: no information  Case 3: heroin and alcohol  Case 4: heroin, BDZ, flupenthixol  Case 5: BDZ | Nystagmus | Case 1: Female. Nystagmus until 3y. Typical development  Case 2: Male. Nystagmus presented at 30mo, still present at 38mo. Noted to have psychomotor disability  Case 3: Male. Nystagmus still present at 8mo. Rest of examination normal  Case 4: Female. Nystagmus at 4mo, resolved by 10mo  Case 5: Male. Nystagmus noted at 2mo, resolved by 9mo | Case 1: NAS, treatment not stated  Case 2: No clarification regarding the developmental delay  Case 3: Preterm (34wk). NAS but treatment not stated  Case 4: NAS but treatment not stated  Case 5: NAS but treatment not stated |
| Hamilton et al.^63^ | C | 20 | n/a | 3mo–7y | No methadone information.  11 out of 20 additionally exposed to BDZ, eight out of 20 to heroin | Full age-appropriate visual assessment  CVI | 95% had reduced visual acuity  70% had nystagmus (horizontal, mostly pendular or jerk)  50% had delayed visual maturation  35% had strabismus  30% had refractive errors.  25% (*n*=5) had significant neurodevelopmental problems (four developmental delay, one cerebral palsy). Incidence of CVI was 25% | Two infants born preterm included 12 treated for NAS, drug not stated. Three children with neurodevelopmental problems had CVI  4 out of 20 had MRI scans, one reported abnormal white matter signal |
| Gupta et al.^64^ | C | 22 | n/a | Mean 18.5mo (range 4mo–56mo) | No methadone information.  6 out of 22 heroin  12 out of 22 BDZ  2 out of 25 significant alcohol | Nystagmus (± compensatory head posture)  Strabismus  Binocular best corrected visual acuity (logMAR)  Other visual abnormalities | All children had horizontal nystagmus: 9 out of 22 jerk nystagmus, 8 out of 22 pendular, 5 out of 22 type nystagmus not stated.  16 out of 22 strabismus, 15 out of 16 esotropia, 1 out of 16 exotropia, 6 no strabismus.  Median visual acuity 0.5 logMAR (range 0.2–1) in 18 children where visual acuity testing possible. One child not fixing and two fixing and following when tested at 4 and 6mo  2 out of 22 bilateral optic nerve hypoplasia, 8 out of 22 delayed visual maturation | Case series of 25 children, two exposed to opiates other than methadone and one exposed only to BDZ  Gestational age at birth not stated. 12 out of 25 demonstrated signs of NAS; information about treatment for NAS not stated |
| Tinelli et al.^65^ | C | 2 | n/a | Case 1: 3mo–12mo  Case 2: 2.5mo–8mo | Case 1: 30  Case 2: 35  MUS | Pendular horizontal nystagmus | Case 1: Preterm (33wk). Nystagmus noted at 2mo, still present at 12mo. Reduced visual acuity (1.3 logMAR) noted at 3mo but gradually improved (0.47 logMAR).  Case 2: Term (40wk). Nystagmus noted at 5mo with associated reduced visual acuity (0.87 logMAR). Nystagmus still present at 8mo but able to follow target. Visual acuity improving (0.47 logMAR) | Case 1: No NAS. Normal cranial ultrasound and fundi at 2mo. At 1y development normal  Case 2: NAS treated with phenobarbital for 15d. Flash visual evoked potential prolonged latency and reduced amplitude right eye. Neurological examination at 8mo normal |
| Konijnenberg et al.^67^ | B | 22 | 25 | Mean age 4y | Mean dose 86.19^j^  Polydrug common: 45% BDZ, 22% intravenous drug use, 18% AMP | Saccade latency  SNP difference score (assessed using eye tracking) | Mean saccade latency(ms) 376.3 (74.6) vs 331.2 (45.50)^k^. SNP difference score: 12 (85.93) vs 23.5 (45.50)^l^.  Less random distractor trials (121 vs 315) and unexposed trials (115 vs 336) included in the analysis^m^ | Part of a study comparing burprenorphine exposed and methadone-exposed infants with an unexposed group, which was not matched for maternal or infant characteristics.  Mean gestational age at birth 38.7wk vs 39.8wk, range not stated. Assessor blinding not stated. 13 out of 22 treated for NAS, drug not stated |
| McGlone et al.^26^ | A | 81^g^ | 26^g^ | 6mo | No methadone dosing information  Polydrug use in 90%: 75% opioids; 67% BDZ; 64% cannabis; 26% stimulants.  20/46 tested for FFAE had elevated levels.  3 out of 18 control infants had elevated FFAE and two tested positive for cannabinoids. | Modified Atkinson Battery^h^ | 32 out of 81 vs 2 out of 26 infants failed the visual assessment^i^.  Abnormalities in methadone-exposed infants:  Strabismus (25%): 12 exotropia, eight esotropia, reduced visual acuity >0.9 logMar (22%), horizontal nystagmus (11%)  Methadone-exposed infants had a fivefold higher chance of failing their visual assessment after correcting for alcohol exposure (*p*=0.007)  Relative risk of abnormal visual assessment in methadone-exposed infants was 5.1 (95% CI 1.3–20), *p*=0.02 | Initial cohort: 100 methadone exposed vs 50 unexposed (follow-up study of same cohort as McGlone et al.^58,70^).  Infants <36wk gestational age excluded. One paediatrician and one optometrist assessed vision. Optometrist blinded to group. Corrected for confounding effect of excess prenatal alcohol exposure  55 out of 81 treated NAS with morphine. No infant had a clinical diagnosis of fetal alcohol syndrome |
| Yoo et al.^66^ | B | 32 | 0 | Infancy; ages not stated; follow-up at various ages | Dose available for 24 out of 32; maximum dose <100 *n*=14, ≥100 *n*=9. Polydrug available for 24/32, MUS; psychotropic medication 75%, heroin or opioid pain medication 71%, cocaine 67%, BDZ 37%, marijuana 37%, alcohol 29%, buprenorphine 4%, barbiturates 4% | Full, age-appropriate ophthalmic exam. Strabismus,  refraction, nystagmus, amblyopia, ocular alignment | 21 out of 32 with strabismus (16 exodeviations, five esodeviations), median age at onset 12mo. 5 out of 21 with strabismus had additional nystagmus. Presence of strabismus not associated with methadone dose, exposure by trimester, polydrug exposure, history of NAS, prematurity, or small for gestational age. | Nine infants born preterm and five small for gestational age infants included  27 NAS, 25 out of 27 treated, drug not stated. Three with perinatal intracranial disease.  Blinding of assessor not stated.  Likely underestimate as sample limited by insurance cover and incomplete follow-up. |

^a^Quality rating: A, good; B, intermediate; C, poor; based on modified Grading of Recommendations Assessment Development and Evaluation criteria (Table SI, online supporting information). ^b^Age expressed in months (mo) or years (y). ^c^Drug information includes mean daily methadone dose (in milligrams), maternal urine screening, and/or infant urine screening for drug exposure and information on maternal polydrug use (defined as methadone plus any other drug use during pregnancy; excluding tobacco), where these are reported. Unless otherwise stated, all information in this column refers to methadone-exposed group only. ^d^Scores are presented as mean values (standard deviation) unless otherwise stated; ^e^Comments include information on attrition, matching, gestation, blinding, proportion of infants treated for neonatal abstinence syndrome (NAS), where provided in the original study. ^f^Flash visual evoked potential categorized as typical, atypical, immature, or not detectable. ^g^Same cohort as McGlone et al.,^58,70^ examined at 6mo. ^h^Atkinson Battery is a full visual assessment and includes pupil response to light, observation for nystagmus, convergence of eyes to approaching object, defensive blink, visual following of falling toy, batting and reaching, near retinoscopy, dynamic retinoscopy, Cardiff acuity card; ^i^Fail defined as presence of strabismus, nystagmus, reduced visual acuity or refractive error >3 dioptres. ^j^Mean methadone dose excludes outlier daily dose of 660mg methadone; ^k^Proportion of infants affected per group is not stated; ^l^No *p* value stated. ^m^Trials excluded if child looked at distractor instead of target, failed to fixate, or anticipated the position of the target before it appeared. MUS, maternal urine screening; NAS, neonatal abstinence syndrome; BDZ, benzodiazepine; CVI, cerebral visual impairment; AMP, amphetamine; SNP, spatial negative priming (SNP effect refers to the longer time it takes to focus on target when it appears in the same place as a distractor has previously appeared in the random distractor trials; in control trials the target appears in a different location, and should be quicker to locate). FFAE, free fatty acyl esters (a biomarker of alcohol consumption in utero).
