# Supplementary figures and images for: Childhood neurodevelopment after prescription of maintenance methadone for opioid dependency in pregnancy: a systematic review and meta‐analysis
Source: Dev Med Child Neurol. 2018 Dec 3;61(7):750–60. doi: 10.1111/dmcn.14117 (PMC6617808; doi:10.1111/dmcn.14117)

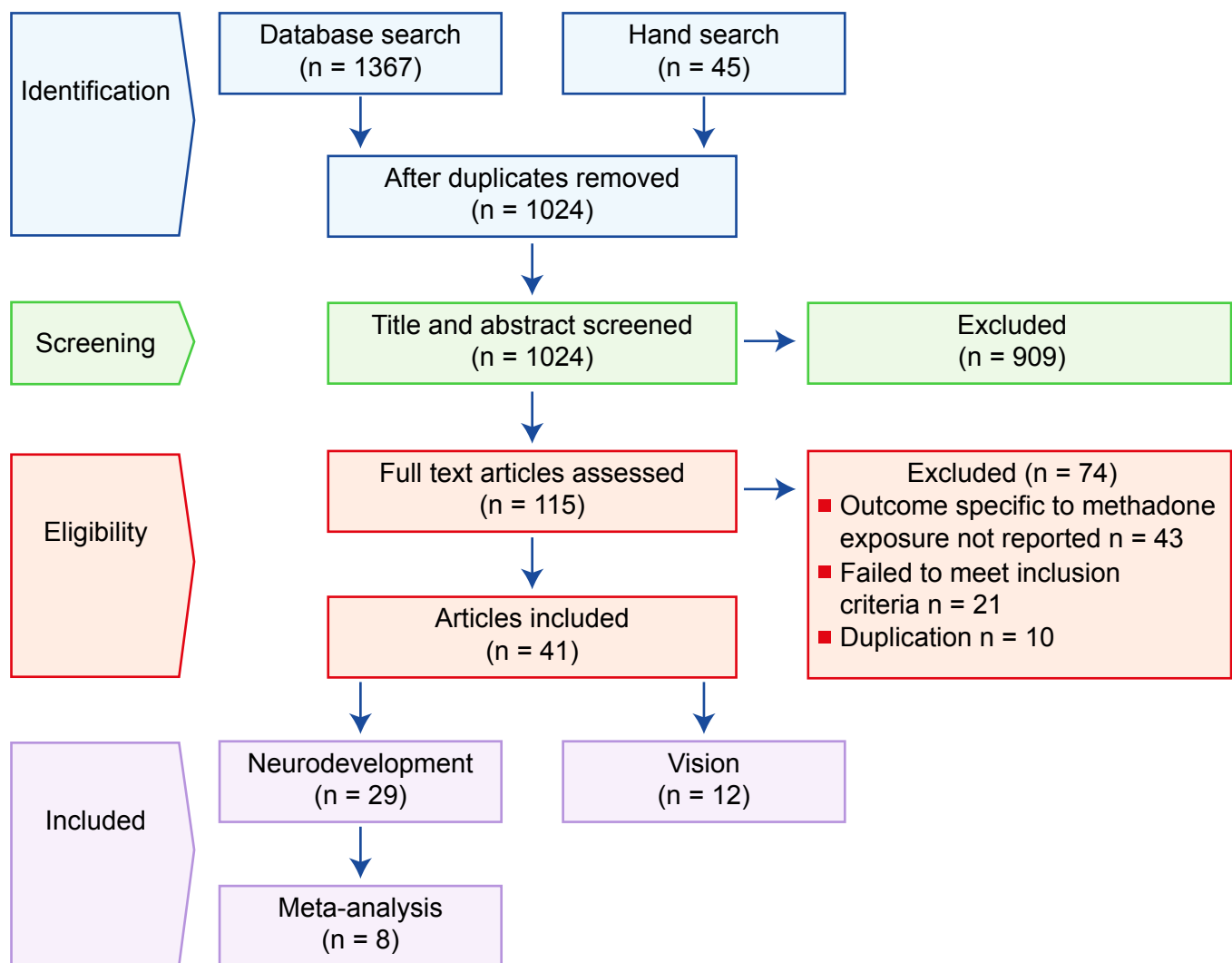

Supplement: Supplementary file 5 — Figure S1: Identification and selection. Preferred Reporting Items for Systematic Reviews and Meta‐Analyses (PRISMA) flowchart showing process of inclusion and exclusion of studies. [file DMCN-61-750-s001.pdf]
